# A Locus Encompassing the Epstein-Barr Virus *bglf4* Kinase Regulates Expression of Genes Encoding Viral Structural Proteins

**DOI:** 10.1371/journal.ppat.1004307

**Published:** 2014-08-28

**Authors:** Ayman El-Guindy, Francesc Lopez-Giraldez, Henri-Jacques Delecluse, Jessica McKenzie, George Miller

**Affiliations:** 1 Department of Pediatrics, Yale University School of Medicine, New Haven, Connecticut, United States of America; 2 Yale Center for Genome Analysis (YCGA), Yale University, West Haven, Connecticut, United States of America; 3 Department of Tumor Virology, German Cancer Research Center, Im Neuenheimer Feld, Heidelberg, Germany; 4 Department of Molecular Biophysics and Biochemistry, Yale University School of Medicine, New Haven, Connecticut, United States of America; 5 Department of Epidemiology and Public Health, Yale University School of Medicine, New Haven, Connecticut, United States of America; University of Wisconsin-Madison, United States of America

## Abstract

The mechanism regulating expression of late genes, encoding viral structural components, is an unresolved problem in the biology of DNA tumor viruses. Here we show that BGLF4, the only protein kinase encoded by Epstein-Barr virus (EBV), controls expression of late genes independent of its effect on viral DNA replication. Ectopic expression of BGLF4 in cells lacking the kinase gene stimulated the transcript levels of six late genes by 8- to 10-fold. Introduction of a BGLF4 mutant that eliminated its kinase activity did not stimulate late gene expression. In cells infected with wild-type EBV, siRNA to BGLF4 (siG4) markedly reduced late gene expression without compromising viral DNA replication. Synthesis of late products was restored upon expression of a form of BGLF4 resistant to the siRNA. Studying the EBV transcriptome using mRNA-seq during the late phase of the lytic cycle in the absence and presence of siG4 showed that BGLF4 controlled expression of 31 late genes. Analysis of the EBV transcriptome identified BGLF3 as a gene whose expression was reduced as a result of silencing BGLF4. Knockdown of BGLF3 markedly reduced late gene expression but had no effect on viral DNA replication or expression of BGLF4. Our findings reveal the presence of a late control locus encompassing BGLF3 and BGLF4 in the EBV genome, and provide evidence for the importance of both proteins in post-replication events that are necessary for expression of late genes.

## Author Summary

Epstein-Barr virus (EBV) is linked to the development of several types of cancer. Synthesis of structural proteins, a group of proteins that forms the protein shell around the viral genome, is essential for EBV infection and pathogenesis. Genes encoding structural proteins are expressed late in the viral life cycle after amplification of the viral genome. The mechanism controlling expression of this group of proteins represents a longstanding conundrum in EBV and other DNA viruses. In this report, we demonstrate that two EBV regulatory proteins control synthesis of mRNAs encoding viral structural proteins. These two proteins are: BGLF4, a protein kinase conserved in all herpesviruses, and BGLF3, a protein of unknown function with no cellular counterparts. We present evidence that the enzymatic activity of BGLF4 is required after replication of viral DNA to stimulate expression of structural proteins. BGLF3 and BGLF4 are expressed from the same locus in the genome; the two proteins work in concert and independently to promote expression of viral genes encoding structural proteins. Our findings provide novel insights into control of expression of genes encoding viral structural proteins. The enzymatic activity of BGLF4 is a potential target for development of new antiviral drugs.

## Introduction

Late genes encode structural proteins necessary for virion assembly. A common theme among DNA viruses is the strict dependence of late gene expression on the onset of viral DNA replication. Disruption of replication, using inhibitors or mutating a replication-essential gene, blocks synthesis of late products. The link between these two processes led to models that focus on genome amplification as the principal regulator of late gene expression. These models propose that changes in DNA modifications such as a decrease in methylation as a result of de novo DNA synthesis, or displacement of viral or cellular repressors bound to elements in late promoters by the replication machinery trigger late gene expression. However, mechanisms that link late gene expression to replication have not been elucidated. Reports in herpesviruses suggest that replication *per se* is not sufficient to activate late gene expression. Pioneering work by Ren Sun and colleagues in murine herpesvirus-68 (MHV-68) identified four early viral proteins, ORF18, 24, 30 and 34, to be required for expression of late genes but dispensable for viral DNA replication [1], [2], [3], [4]. Homologs of ORFs 18, 24 and 34 in human cytomegalovirus (hCMV) map to three unique long (UL) sequences 79, 87 and 95, respectively [5], [6]. The function of the MHV68 and hCMV proteins in activating late gene expression has not been elucidated. In Epstein-Barr virus (EBV), the only protein so far characterized as essential for activation of late genes and not DNA replication is BcRF1, a homolog of ORF24 in MHV-68 and UL87 in CMV [7]. BcRF1 is a TATA box binding-like protein that specifically binds to a non-canonical TATA element (TATT) present in most late promoters [8], [9].

Viral late promoters differ from viral promoters of other kinetic classes and cellular promoters that rely on transcription factor-binding sites located upstream of the TATA box. Activation of late promoters is primarily dependent on a distinct TATA box and a downstream initiator sequence that spans the transcription start site (TSS) [9], [10], [11]. Involvement of upstream elements in regulation of late promoters is postulated to modulate transcription efficiency [12], [13], [14], [15].

BGLF4 is the only Ser/Thr protein kinase encoded by EBV [16]. Homologs of BGLF4 are conserved among the herpesvirus family [17]. These conserved human herpesvirus protein kinases (CHPKs) share sequence and positional similarities, but exhibit both unique and overlapping substrate specificities [18], [19]. BGLF4, like other CHPKs, functionally mimics cellular cyclin-dependent kinases but displays broader substrate specificity [17], [19], [20], [21], [22]. For instance, protein array phosphorylation experiments identified 21 EBV proteins as putative substrates of BGLF4; half of these proteins are shared targets with CDK1/cyclinB [23]. A number of cellular cyclin-dependent kinase substrates are also modified by BGLF4 such as pRB, p27, condensin, MCM4, stathmin, elongation factor 1 delta, and nuclear lamin A/C [19], [20], [21], [22], [24], [25], [26], [27], [28], [29]. Phosphorylation of lamin A/C, another CDK substrate [30], by BGLF4 causes dissolution of the nuclear lamina, a step considered essential for egress of viral capsids from the nucleus [27], [31], [32].

BGLF4 has been extensively investigated to determine its potential role in viral DNA replication. Expression of BGLF4 occurs during the early phase of the lytic cycle and reaches maximal levels following viral DNA replication [33]. BGLF4 localizes to replication compartments, a site of viral DNA replication and late gene expression [34], [35]. BGLF4 modulates gene expression by its capacity to phosphorylate transcription factors, histones and chromatin modifying enzymes, such as BMRF1, ZEBRA, EBNA2, EBNA-LP, IRF3, UXT, HDAC1, H1, and TIP-60 [16], [36], [37], [38], [39], [40], [41], [42], [43], [44], [45]. However, the evidence in favor of a role of BGLF4 in regulating early gene expression and viral DNA replication during the EBV lytic cycle is conflicting. Abolishing expression of BGLF4 had no significant effect on early gene expression [46] and had little (<2-fold) to no impact on viral DNA replication [28], [31], [32], [44], [45], [47]. Perhaps the most established phenotype for disrupting the expression or the activity of BGLF4 is the significant reduction in the amount of virus released by lytic infected cells [27], [31], [48]. This phenotype is consistent with a crucial role for BGLF4 in events occurring after viral DNA replication.

In this study, we employed knockout and knockdown approaches to thoroughly investigate the role of BGLF4 in regulating lytic viral gene expression and its correlation with DNA replication. We found that the kinase activity of BGLF4 is necessary for expression of late genes but not viral DNA replication. Using RNA-seq analysis we demonstrated that expression of most late genes is reduced in the absence of BGLF4. We identified a late control locus encompassing BGLF4 and BGLF3. Knockdown of BGLF3 abolished late gene expression independent of any effect on the level of BGLF4 or viral DNA replication. Our findings identify two EBV genes as regulators of late gene expression and establish the presence of additional checkpoints beyond viral DNA replication that are necessary for expression of EBV structural proteins.

## Results

### BGLF4 significantly up-regulates expression of the late BFRF3 protein

**Figure 1:**
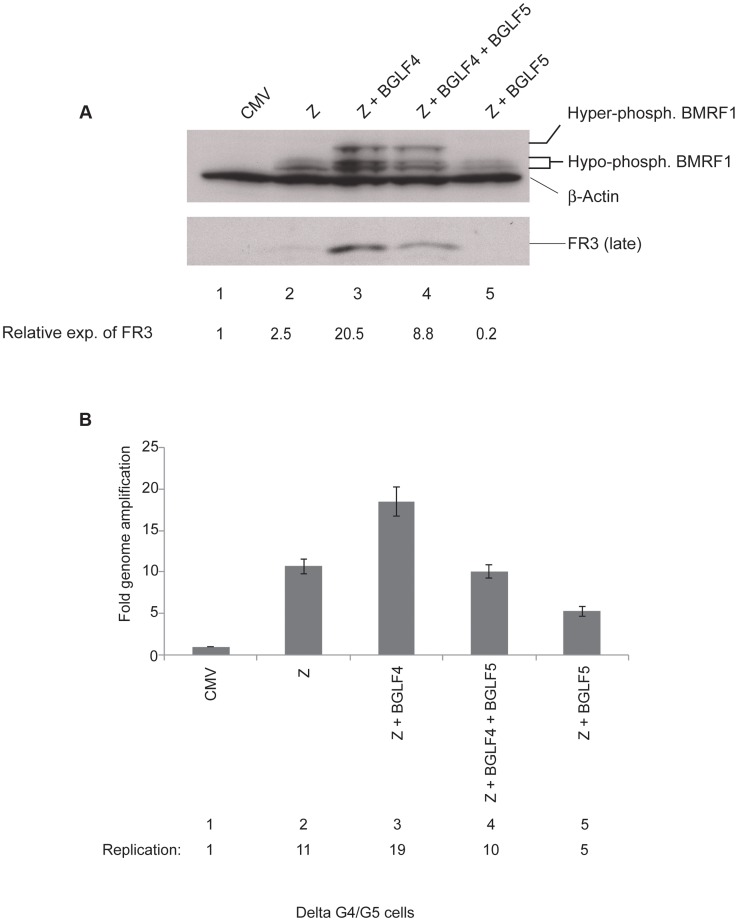
BGLF4 markedly enhances expression of the BFRF3 late protein. The experiment illustrates the effect of introducing BGLF4 and BGLF5 on expression of EBV lytic proteins and viral DNA replication in delta G4/G5 cells. (A) Western blot analysis of EBV early and late proteins expressed 48 h after transfection of the indicated plasmids. (B) Quantitative PCR measuring amplification of the EBV genome from the same samples as in panel A. Z, ZEBRA; FR3, BFRF3 minor capsid protein (late); BMRF1, DNA polymerase processivity factor (early).

BGLF4 is transcribed as a multicistronic message that also encodes BGLF5, a DNA alkaline exonuclease involved in host cell shutoff [47], [49]. To study the role of BGLF4 in regulating the lytic cascade we used delta G4/G5 cells, which are 293 cells harboring BGLF4/BGLF5-null EBV bacmid virus [47]. Cells were harvested 48 h after transfection to allow for analysis of viral DNA replication and late gene expression. Typically, transient expression of ZEBRA in EBV positive cells induces expression of three major kinetic classes of lytic genes, these are: very early, early and late genes [50], [51]. In delta G4/G5 cells, we found that ZEBRA activated the EBV lytic cycle as demonstrated by expression of the early *bmrf1* gene encoding the DNA polymerase processivity factor (Fig, 1A lane 2). However, expression of the late BFRF3 protein (FR3), the minor viral capsid protein, was noticeably low, only 2.5-fold higher in cells transfected with ZEBRA relative to empty vector (CMV) (Fig. 1A, lanes 1 and 2). Complementation with BGLF4 significantly enhanced the level of FR3 to 20.5-fold relative to empty vector and 8.2-fold relative to ZEBRA-transfected cells (Fig. 1A, lane 3). Complementation with BGLF5 did not enhance expression of FR3, a result suggesting that the effect on late gene expression is specific to BGLF4. BMRF1 is a *bona fide* substrate of the BGLF4 kinase [16], [36], [52]. Therefore, hyper-phosphorylation of BMRF1 is a marker for the intracellular kinase activity of BGLF4. Hyper-phosphorylated BMRF1 was only detected in lytic cells transfected with the BGLF4 expression vector (Fig. 1A, compare lanes 2 and 5 with 3 and 4). The experiment was repeated to confirm that BGLF4, and not BGLF5, reproducibly up-regulated expression of FR3 when expressed alone or together with BGLF5 (Fig. S1).

Since FR3 is a late protein, we examined the possibility that lack of BGLF4 might disrupt viral DNA replication and thus compromise expression of FR3. To assess the effect of BGLF4 on the extent of viral genome amplification we prepared genomic DNA from aliquots of the same cells that were examined for protein expression. The relative concentration of viral DNA was assessed using primers specific to the upstream region of oriLyt and quantitative polymerase chain reaction (qPCR). We found that expression of BGLF4 slightly increased viral DNA replication by 1.8-fold relative to cells transfected with ZEBRA alone or with ZEBRA plus BGLF4 and BGLF5 (Fig. 1B, lanes compare lane 3 with 2 and 4). Over-expression of ZEBRA and BGLF5 reduced the extent of EBV genome amplification by 50% relative to ZEBRA alone (compare lanes 2 and 5). Thus lack of BGLF4 lead to a modest reduction (1.8-fold) in viral DNA replication but caused a more pronounced defect (8.2-fold) on synthesis of FR3 late protein.

### The kinase activity of BGLF4 is necessary for activation of late gene expression but not viral DNA replication

Lysine 102 in BGLF4 corresponds to a conserved lysine present in the catalytic domain of protein kinases [53]. Substitution of lysine 102 to isoleucine abolishes the kinase activity of BGLF4 [20]. To determine whether the kinase activity of BGLF4 is required for its effects on viral DNA replication and synthesis of FR3, we expressed BGLF4 or BGLF4(K102I) together with ZEBRA in delta G4/G5 cells and harvested the cells after 48 h. We did not detect any difference in viral DNA replication as a result of expressing the kinase-active or -inactive forms of BGLF4. Both wild-type and mutant BGLF4 equally enhanced viral DNA replication by 2.6-fold relative to ZEBRA alone (Fig. S2B, lanes 2, 4, and 6). However a clear difference was observed between BGLF4 and BGLF4(K102I) in promoting expression of the FR3 late gene (Fig. S2A). Wild-type BGLF4 increased expression of FR3 by 9-fold whereas the kinase dead mutant of BGLF4 enhanced the level of FR3 by 1.66-fold. Wild-type and mutant forms of BGLF4 protein were equally expressed. The level of BMRF1 protein did not change when the kinase activity of BGLF4 was disrupted (Fig. S2A), but the hyper-phosphorylated form of BMRF1 was only detected in cells expressing active BGLF4 and not the kinase dead mutant. These findings show that the kinase activity of BGLF4 is necessary for enhanced expression of FR3 but is not required for the low level of stimulation of viral DNA replication by BGLF4.

### Increasing concentrations of wild-type BGLF4, but not the kinase dead mutant, up-regulate expression of the late FR3 protein independent of effects on viral DNA replication

**Figure 2:**
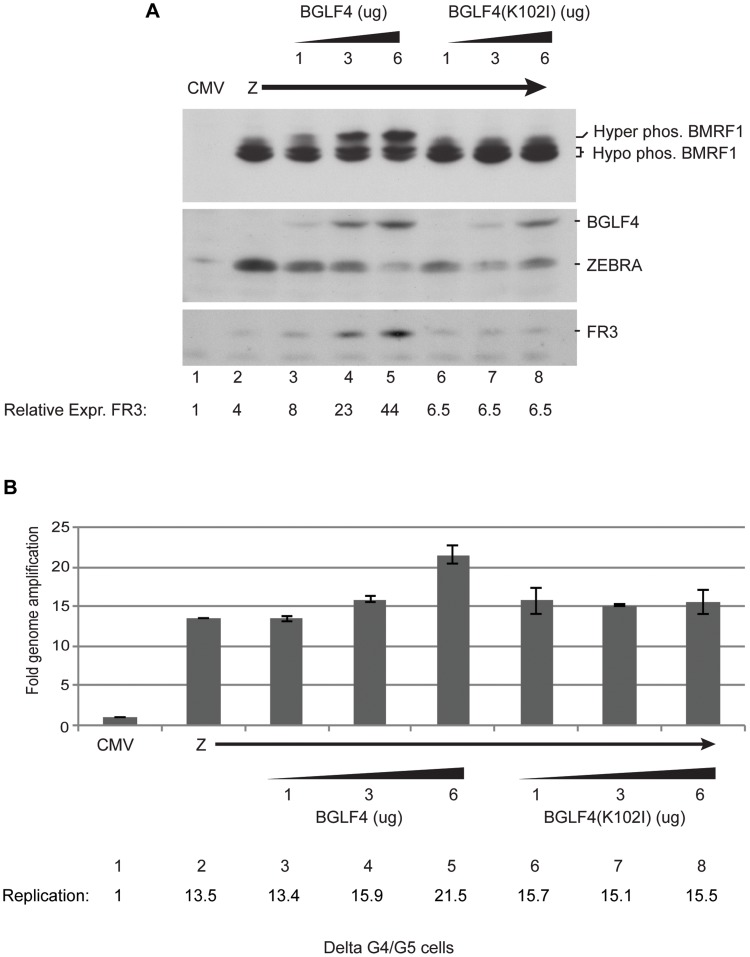
Increasing concentrations of wild-type BGLF4, but not the kinase dead mutant, enhances late gene expression. Delta G4/G5 cells were transfected with ZEBRA alone or together with 1, 3 and 6 µg of wild-type or kinase dead mutant BGLF4. Cells harvested after 48 h were divided into two aliquots to study expression of lytic proteins and viral replication. (A) Western blot analysis to determine the expression levels of BMRF1, BGLF4, ZEBRA and FR3 proteins using specific antibodies. (B) Quantitative measurement of the extent of viral DNA replication using real time PCR.

The previous experiment suggested that the kinase activity of BGLF4 is necessary for up-regulating expression of the late FR3 protein. To further examine this point, we expressed increasing concentrations of wild-type or mutant BGLF4 in the presence of a constant amount of ZEBRA. After 48 h, transfected delta G4/G5 cells were harvested and the levels of FR3, BMRF1, BGLF4 and ZEBRA proteins were assessed using Western blot analysis. By comparing the effect of the two forms of BGLF4, we found that as we increased the level of wild-type BGLF4 there was a progressive increase in the amount of FR3 protein expressed. The increase in the amount of BGLF4 protein expressed was associated with an increase in the level of hyper-phosphorylated form of BMRF1 (Fig. 2A). On the contrary, in cells expressing increasing levels of the kinase inactive form of BGLF4, expression of FR3 remained constant at a low level and BMRF1 was not hyperphosphorylated.

Using qPCR we studied the effect of expressing various concentrations of BGLF4 and BGLF4(K102I) on viral DNA replication; we prepared DNA from the same cells used in figure 2A. We only detected a change in the amount of viral DNA when we transfected 6 µg of wild-type BGLF4. At this input, replication increased by 1.6-fold relative to expression of ZEBRA alone (compare lanes 2 and 5). Findings from Figures S2 and 2 strongly indicate that the effect of BGLF4 on late gene expression is kinase dependent and is separate from its effect on viral DNA replication.

### BGLF4 regulates late genes transcripts

**Figure 3:**
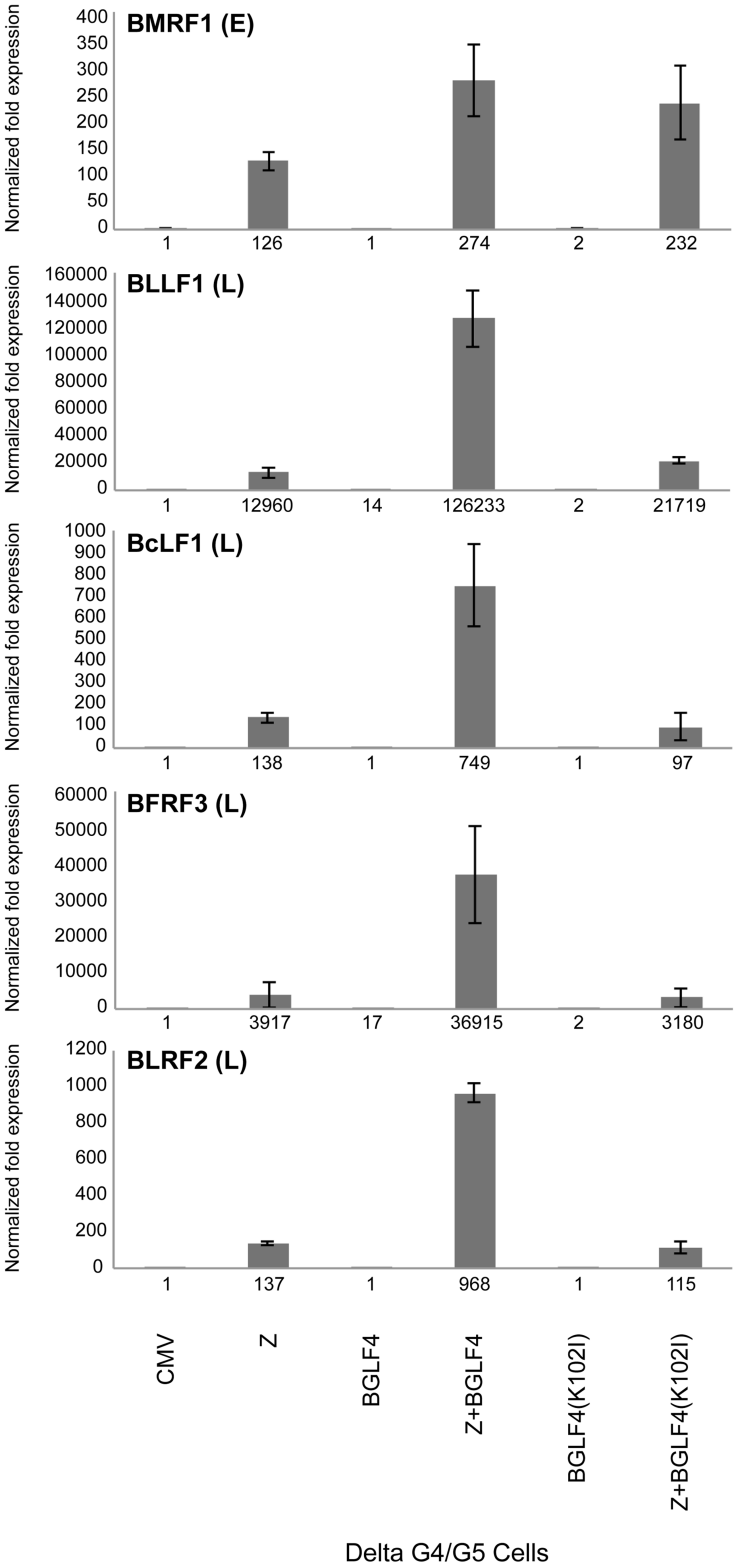
Expression of BGLF4 significantly up-regulates the level of late transcripts. The abundance of five EBV lytic transcripts was assessed, using quantitative RT-PCR, in delta G4/G5 cells transfected with the following plasmids: empty vector (CMV), ZEBRA (Z), BGLF4, ZEBRA plus BGLF4, mutant BGLF4(K102I), and ZEBRA plus mutant BGLF4(K102I). BMRF1 encodes an early transcript encoding the DNA polymerase processivity factor. BFRF3, BcLF1, BLLF1 and BLRF2 are late transcripts that encode the minor viral capsid, the major capsid protein, gp350/220 major glycoprotein, and a tegument protein, respectively. The level of each of the five transcripts was normalized to empty vector control.

In the next experiment we asked whether the effect of BGLF4 on expression of FR3 could be detected at the transcript level, and whether BGLF4 regulated mRNA expression of other late genes. We prepared total RNA from fractions of the same samples that were previously used to generate figure S2 and employed quantitative reverse transcriptase polymerase chain reaction (RT-qPCR) to assess the level of five lytic transcripts. Four of these EBV transcripts, encoding the major and minor capsid proteins (BcLF1 and BFRF3), the major glycoprotein gp350/220 (BLLF1), and a tegument protein (BLRF2), have late kinetics and one, encoding BMRF1, has early kinetics. When co-expressed with ZEBRA, wild type BGLF4 or BGLF4(K102I) enhanced the level of BMRF1 by 2.17 and 1.8-fold, respectively (Fig. 3). This result suggested that BGLF4 had a limited stimulatory effect on expression of the early BMRF1 transcript in a manner independent of its kinase activity. However, the kinase activity of BGLF4 was necessary for efficient expression of the four late transcripts examined. The level of all four late transcripts was less in delta G4/G5 cells expressing ZEBRA alone or ZEBRA plus G4(K102I) compared to co-expression of ZEBRA and wild-type BGLF4 (Fig. 3). Expression of late transcripts was reduced by an average of 7.9-fold (range 9.7 to 5.4) in cells expressing ZEBRA alone and a mean of 8.4-fold (range 11.6 to 5.8) in cells expressing ZEBRA plus the kinase dead mutant. These results indicate that the kinase activity of BGLF4 markedly up-regulates expression of late but not early transcripts.

### Knockdown of BGLF4 abolished expression of the late BFRF3 protein

**Figure 4:**
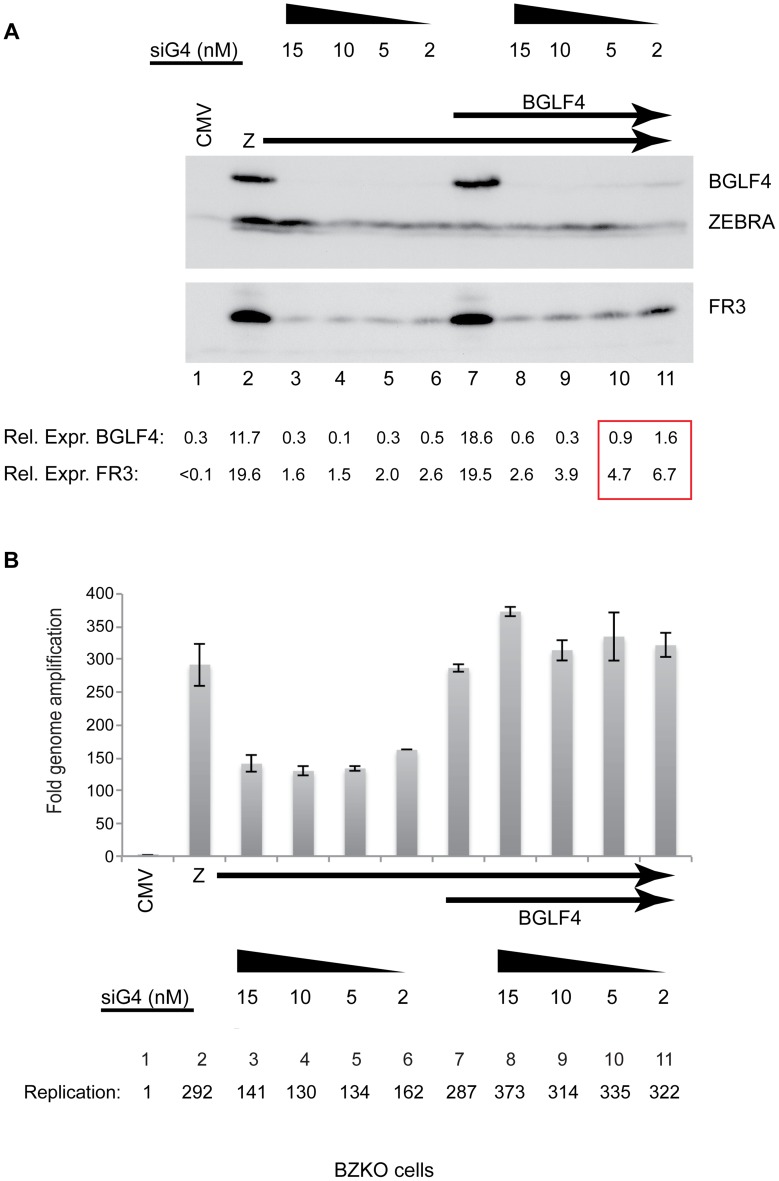
siRNA specific to BGLF4 abolishes expression of the late FR3 protein. (A) Immunoblot of BZKO cell lysates expressing ZEBRA alone (lanes 2 to 6) or ZEBRA and BGLF4 (lanes 7 to 11) together with increasing concentrations (2 to 15 nM) of siRNA complimentary to BGLF4 (siG4). The levels of BGLF4 and late BFRF3 (FR3) proteins were determined by densitometry. (B) Fold change in viral lytic DNA replication as a result of transfecting siRNA specific to BGLF4 (siG4). Total DNA was prepared from the same cells used in the immunoblot in panel A.

To eliminate the possibility that the markedly enhanced synthesis of EBV late products in delta G4/G5 cells following ectopic expression of BGLF4 was due to its overexpression, we used siRNA to knockdown endogenous BGLF4. For these experiments we used BZKO cells, which are 293 cells containing an EBV bacmid that lacks a functional gene for ZEBRA, to study the effect of silencing BGLF4 on viral DNA replication and late gene expression. Transfection of ZEBRA (2 µg) induced the lytic cycle and activated expression of endogenous BGLF4 (Fig. 4A, lane 2). To knock down expression of BGLF4 we co-transfected four concentrations, 2, 5, 10 and 15 nM, of siRNA specific to the viral protein kinase (siG4) (Fig. 4A, lanes 3 to 6). Cells were harvested after 48 h and expression of the FR3 protein was assessed using Western blot analysis. We found that all four concentrations of siG4 abolished expression of BGLF4. The reduction in the level of the late FR3 protein correlated with the amount of siG4 used to knockdown expression of BGLF4. This reduction in the level of the FR3 protein ranged between 9.8- to 12.3-fold relative to lytic cells without siG4 (Fig. 4A, lanes 3 to 6). We used qPCR to assess the effect of abolishing expression of BGLF4 on replication of the EBV genome. Knocking down of endogenous BGLF4 reduced EBV DNA levels by 2.1- to 2.4-fold (Fig. 4B, compare lane 2 with lanes 3 to 6). The effect of siRNA to BGLF4 on replication was similar in magnitude to that observed in previous experiments using delta G4/G5 cells in which expression of either wild-type or the kinase dead mutant of BGLF4 enhanced expression of BMRF1 and viral DNA replication by ∼2-fold (Figs. 1, S2 and 2).

To attempt to segregate the effect of BGLF4 on viral DNA replication from that on late gene expression we gradually increased the level of BGLF4 expressed by titrating the amount of siG4 (15, 10, 5 and 2 nM) co-transfected together with fixed concentrations of plasmids encoding ZEBRA (2 µg) and BGLF4 (3 µg). At the highest concentration of siG4 (15 nM), the level of the FR3 protein was markedly reduced by 7.5-fold compared to cells without siG4 (Fig. 4A, compare lanes 7 and 8). However, the level of viral DNA in the same cells was not compromised as a result of knocking down BGLF4; on the contrary the amount of viral DNA present was slightly higher relative to cells expressing ZEBRA alone or ZEBRA plus BGLF4 (Fig. 4B, lanes 8–11). As the level of BGLF4 expressed increased due to decreasing the amount of siG4 transfected, there was a proportional increase in the amount of the FR3 late protein, but the level of viral DNA replication was unaffected. These results establish a direct relationship between the level of BGLF4 and expression of the late FR3 protein independent of any effect of BGLF4 on viral DNA replication.

### Silent mutations in BGLF4 suppress the effect of siG4 on late gene expression

**Figure 5:**
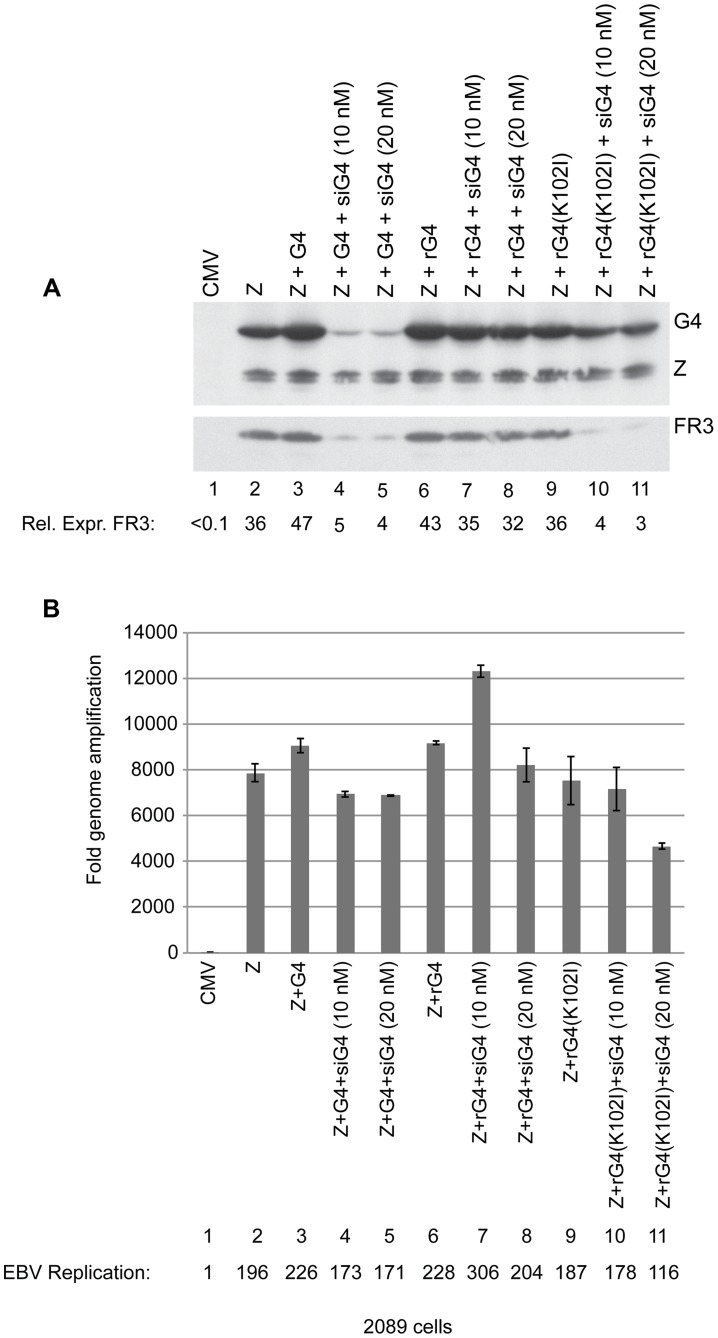
Silent mutations in BGLF4 override the effect of siG4 on late gene expression. (A) Western blot analysis of 2089 cell lysates transfected with the indicated expression vectors. Two concentrations of siRNA specific to BGLF4 (siG4), 10 and 20 nM, were co-transfected with expression vectors encoding ZEBRA and wild-type BGLF4 or kinase dead BGLF4(K102I) (lanes 4, 5, 10 and 11). To demonstrate the specificity of siG4, silent mutations were introduced into the kinase active (rG4) and kinase dead (rG4(K102I)) *bglf4* genes that rendered the BGLF4 transcript resistant to siG4. Lysates of cells harvested 48 h after transfection were analyzed by immunoblotting using antibodies specific to BGLF4, ZEBRA and the late BFRF3 protein (FR3). (B) Quantitative PCR to measure the extent of EBV genome amplification in each condition.

Specificity of siRNA represents a major concern in this widely used approach to down-regulate gene expression. To determine whether the effects of siG4 on late gene expression were a result of down-regulating BGLF4 or an off-target effect, we generated silent mutations in the *bglf4* gene that abolished complementarity between the BGLF4 transcript and siG4 without altering the amino acid sequence. Transfection of siG4 is expected to eliminate expression of endogenous BGLF4 and late genes. However, providing the siG4-resistant BGLF4 ectopically should restore expression of late genes, only if the effect of siG4 is target specific. Two forms of the siG4-resistant BGLF4 were generated, kinase active and kinase dead, referred to as rG4 and rG4(K102I), respectively. To study the effect of siG4 on expression of rG4 and rG4(K102I), we transfected 2089 cells, 293 cells containing wild-type EBV bacmid, with two concentrations of siG4 (10 and 20 nM) together with expression vectors of ZEBRA and rG4 or rG4(K102I). Expression of ZEBRA by itself in 2089 cells triggered the lytic program including expression of the endogenous BGLF4 (Fig. 5A, lane 2). Both concentrations of siG4 were sufficient to abolish expression of ectopic and endogenous BGLF4 in cells co-transfected with ZEBRA and BGLF4 (Fig. 5A, lanes 4 and 5). Expression of rG4 and rG4(K102I) was not affected by the same concentrations of siG4 (Fig. 5A, lanes 7, 8, 10 and 11). This outcome allowed us to assess the specificity of siG4 by restoring expression of BGLF4 and studying its effect on late gene expression. If siG4 non-specifically abolished expression of other proteins that are crucial for late gene expression, then restoring expression of BGLF4 by rG4 would not be sufficient to rescue expression of FR3. We found that expression of rG4 and ZEBRA in the presence of siG4 restored expression of FR3 to normal levels (lanes 7 and 8). However, providing rG4(K102I) instead of rG4, failed to restore the level of FR3 protein (lanes 10 and 11). These findings showed that down-regulation of FR3 in cells transfected with siG4 is due to abolishing expression of BGLF4 with intact kinase activity. Lane 9 shows that cells expressing the transfected kinase dead protein and endogenous BGLF4 are permissive for expression of FR3. While rG4 restored late gene expression, neither rG4 nor rG4(K102I) had any significant effect on viral DNA replication (Fig. 5B).

**Figure 6:**
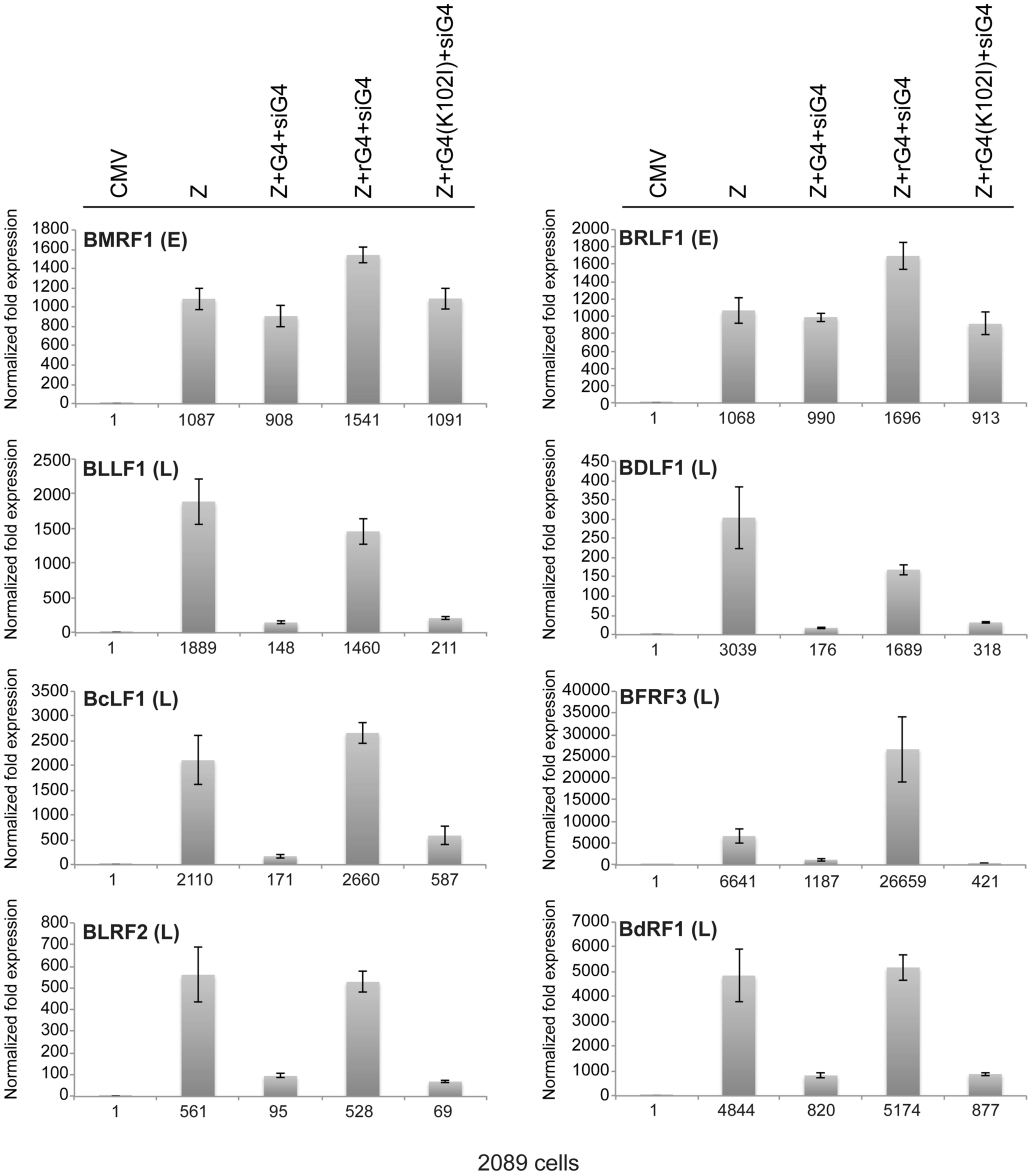
Expression of siG4-resistant BGLF4 restores synthesis of EBV late transcripts. The effect of siG4 on the level of two early and six late viral transcripts was analyzed by RT-qPCR. The early transcripts are BMRF1 (polymerase processivity factor) and BRLF1 (transcription factor). The late transcripts are BLLF1 (gp350/gp220 major glycoprotein), BFRF3 (minor capsid protein), BLRF2 (tegument protein), BDLF1 (triplex capsid protein), BdRF1 (scaffold protein), and BcLF1 (major capsid protein). The relative abundance of each transcript was calculated using the ΔΔ*C_T_* method.

We studied the effect of silencing BGLF4 on the level of eight lytic transcripts in 2089 cells expressing ZEBRA either alone or together with rG4 or rG4(K102I) (Fig. 6). Two of these transcripts, BRLF1 and BMRF1, are synthesized prior to viral DNA replication. The *brlf1* gene encodes the Rta protein, a transcription activator and a replication protein, and the *bmrf1* gene codes for the polymerase processivity factor. Both proteins play vital roles in the process of viral DNA replication [54], [55]. The other six transcripts, with late kinetics, encode the following proteins: major glycoprotein gp350/220 (BLLF1), major capsid protein (BcLF1), tegument protein (BLRF2), triplex capsid protein (BDLF1), scaffold protein (BdRF1), and minor capsid protein (BFRF3). Knockdown of endogenous and transfected BGLF4 by siG4 reduced the level of late transcripts by a median fold-change of 9.1. Conversely, expression of rG4 restored the amount of late transcripts to a level equivalent to that observed in cells transfected with ZEBRA alone. However, expression of rG4(K102I) failed to up-regulate the level of all six late transcripts and resulted in a median reduction of 8.6-fold relative to ZEBRA alone. These results demonstrate a specific role for the kinase activity of BGLF4 in regulating the level of several late viral transcripts.

### Analysis of the EBV transcriptome during lytic induction reveals the general importance of BGLF4 in regulation of late gene expression

**Figure 7:**
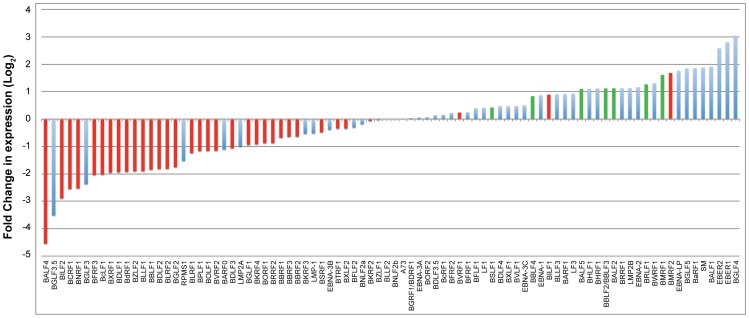
Impact of knockdown of BGLF4 on the EBV transcriptome. RNA-seq analysis compared EBV transcripts expressed in 2089 cells transfected with ZEBRA versus ZEBRA plus siG4. RNA was prepared at 48 h after transfection. The plotted graph was generated by dividing the number of normalized reads of a particular transcript in the presence of siG4 by the number of reads obtained in the absence of siG4. This ratio represents the effect of BGLF4 on the abundance of a particular transcript and is presented as a log base 2-fold. A negative fold-change indicated dependency on BGLF4 for expression. Late genes illustrated in red bars while latent and other lytic genes were illustrated in blue bars.

To study the effect of BGLF4 on the EBV transcriptome during lytic induction, we performed deep sequencing analysis on RNA purified from 2089 cells transfected with empty vector, ZEBRA or ZEBRA plus siG4. To demonstrate that siG4 had silenced BGLF4 in this particular experiment we assessed the levels of BGLF4 and FR3 proteins by Western blot. As expected, ZEBRA induced expression of BGLF4 and FR3; addition of siG4 abolished expression of BGLF4 and the late FR3 protein (data not shown). We used the Expectation-Maximization (EM) algorithm in RSEM to estimate gene expression levels and EBSeq to identify differentially expressed genes [56], [57]. Figure 7 compares the number of normalized reads corresponding to each viral transcript expressed in the absence and presence of BGLF4. The change in expression is represented as a log base 2-fold. Red bars designate late genes; blue bars designate latent and early kinetic classes of lytic genes. Determination of the kinetic classes of EBV genes was based on viral gene classification reported by Yuan et al [58]. Viral transcripts with a negative fold-change represent EBV genes whose expression is BGLF4-dependent, and vice versa. The most striking result was that silencing of BGLF4 down-regulated the level of thirty-one late transcripts. The level of only three late transcripts, encoding BVRF1, BILF1 and BMRF2, increased by 1.2-, 1.8-, and 3.2-fold, respectively (Fig. 7). BVRF1 is a tegument protein and is a homolog of HSV UL25 (cork), BILF1 is a virally encoded G protein coupled receptor, and BMRF2 is a transmembrane envelope protein [59], [60], [61]. siRNA to BGLF4 did not reduce the level of transcripts encoding components of the replication machinery. Silencing of BGLF4 impaired expression of two lytic transcripts of unknown kinetic class, namely, BGLF3.5 and BGLF3 (Fig. 7) [58]. The reduction in the level of these two transcripts in lytic cells expressing BGLF4 compared to cells without BGLF4 was 11.6-fold for BGLF3.5 and 7.3-fold for BGLF3. The functions of BGLF3.5 and BGLF3 in the EBV lytic cycle remain to be characterized. However, *orf34*, a homologue of BGLF3 encoded by murine gamma herpesvirus-68 (MHV-68), is an early gene that was found to be essential for late gene expression and not viral DNA replication [4]. These findings demonstrated that BGLF4 globally enhanced expression of EBV late genes.

### Knockdown of BGLF4 down-regulates expression of BGLF3 and BGLF3.5

RNA-seq analysis showed that silencing of BGLF4 reduced expression of BGLF3 and BGLF3.5. We sought to validate the effect of siG4 on the level of transcripts encoding BGLF3 and BGLF3.5 using RT-qPCR. The data presented in figure S3 represent the mean of two biological replicates. Similar to our observations with the RNA-seq analysis, we found that expression of the *bglf3* and *bglf3.5* genes was reduced in cells lacking BGLF4 by 6-fold and 22-fold, respectively.

**Figure 8:**
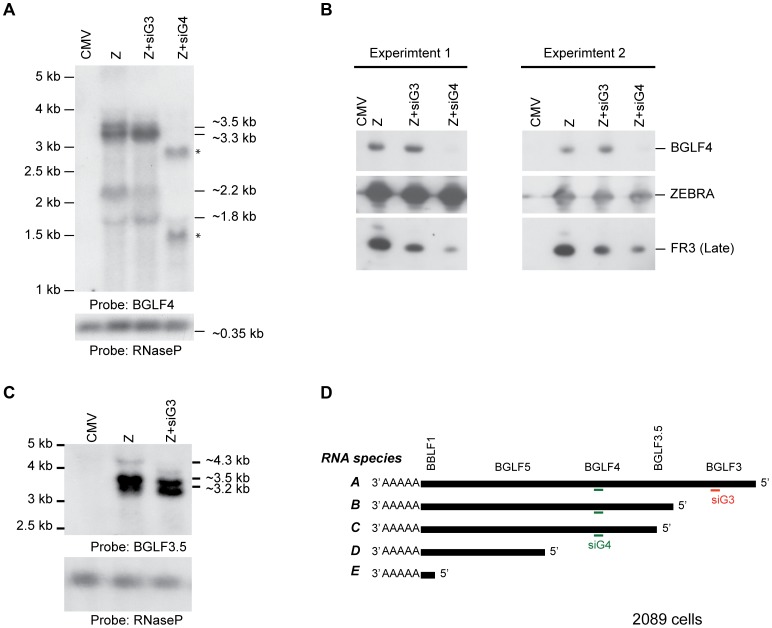
Knockdown of BGLF3 has no effect on expression of BGLF4. To examine the effect of silencing BGLF3 expression on the mRNA and protein levels of BGLF4, we transfected 2089 cells with ZEBRA alone or together with siG3 or siG4. Cells were harvested after 48 h. RNA and protein samples were prepared for Northern (A and C) and Western (B) blot analyses. In cells transfected with ZEBRA, we detected four RNA species: 3.5, 3.3, 2.2 and 1.8 kb using the BGLF4 probe (A). The 4.3 kb was only detected using a probe unique to BGLF3.5 (C). Except for the 4.3 kb, the same RNA species were detected when siG3 was co-transfected. However, the 3.5, 3.3 and 2.2 kb RNA species disappeared in the sample co-transfected with siG4. These RNAs were replaced with two new species approximately 1.6 and 3 kb in size. Examining protein expression in the same samples demonstrated that siG4 and siG3 markedly reduced expression of the late FR3 protein (Fig. 8B). Nonetheless, similar to Fig. 9, siG3 had no effect on the level of the BGLF4 protein. The experiment was repeated twice and similar results were obtained. (D) A schematic diagram of the various transcripts expected to be synthesized from the late control locus. CMV, empty vector; Z, ZEBRA; siG3, siRNA to BGLF3; siG4, siRNA to BGLF4. The asterisks denote probable cleavage products.

The effect of siG4 on BGLF3 and BGLF3.5 could be attributed to two different mechanisms: siRNA to BGLF4 simultaneously reduces the BGLF3 and BGLF3.5 transcripts; BGLF4 regulates synthesis or stability of BGLF3 and BGLF3.5 mRNAs. The *bglf4*, *bglf3.5* and *bglf3* genes are located in a transcription unit with a single poly(A) signal (Fig. 8D). Previous reports suggested that transcripts synthesized from this region are co-terminal [33], [62]. To assess the number and size of transcripts expressed from this locus we purified RNA from 2089 cells transfected with ZEBRA. Using Northern blot analysis and probes directed to the unique sequences in BGLF4 and BGLF3.5 we detected five RNAs that were 4.3, 3.5, 3.3, 2.2 and 1.8 kb long (Fig. 8A and C). Based on the lengths of the genes and the distribution of TATA boxes present in this locus, the 4.3 kb RNA correlates with transcript A (BGLF3-BGLF3.5-BGLF4-BGLF5-BBLF1). siRNA to BGLF3 abolished the 4.3 kb transcript without affecting the level of the other transcripts (Fig. 8C). This result demonstrates that the 4.3 kb RNA is the only transcript that contains the BGLF3 sequence, which is consistent with the composition of transcript A. The 3.5 kb RNA corresponds with transcript B (BGLF3.5-BGLF4-BGLF5-BBLF1); the 3.2 kb RNA with transcript C (BGLF4-BGLF5-BBLF1), and the 1.8 kb RNA with transcript D (BGLF5-BBLF1) (Fig. 8D). The 1.8 kb RNA was detected by the BGLF4 probe presumably as a result of containing BGLF4 sequence in the 5′ UTR of RNA species D (Fig. 8A). A 2.2 kb RNA was only detected using the BGLF4 probe (Fig. 8A); its size does not correlate with any of the predicted RNA transcripts.

siRNA to BGLF4 abolished four RNA species: 4.3, 3.5, 3.3 and 2.2 kb. This outcome conforms to the predicted composition of transcripts A, B, and C, in which all three transcripts contained the BGLF4 sequence (Fig. 8D). Thus, targeting BGLF4 with siG4 silences expression of BGLF3.5 and BGLF3 as well. Transfection of siG4 resulted in two new RNA species that were 1.6 and 3 kb long and were only detected by the BGLF4 probe (Fig. 8 A). The appearance of these two RNAs is consistent with the capacity of siG4 to trigger cleavage of the BGLF4 containing transcripts.

To determine whether BGLF4 has the capacity to increase the level of BGLF3 mRNA either by up-regulating its expression or enhancing the stability of its mRNA, we compared the level of BGLF3 mRNA in 2089 cells transfected with ZEBRA, ZEBRA plus siG4 or ZEBRA plus siG4 and rG4 (siG4 resistant BGLF4). Addition of siG4 reduced the level of BGLF4 and BGLF3 transcripts expressed from the endogenous viral genome. Ectopic expression of rG4 increased the level of BGLF3 transcript by 4-fold relative to cells transfected with ZEBRA plus siG4 (Fig. S4).

These results suggest that siG4 reduces the level of BGLF3 mRNA by two different mechanisms; it directly silences expression of BGLF3, and it knocks down expression of BGLF4, a protein that augments expression or stability of the BGLF3 transcript.

### Knockdown of BGLF3 abolished expression of EBV late genes

**Figure 9:**
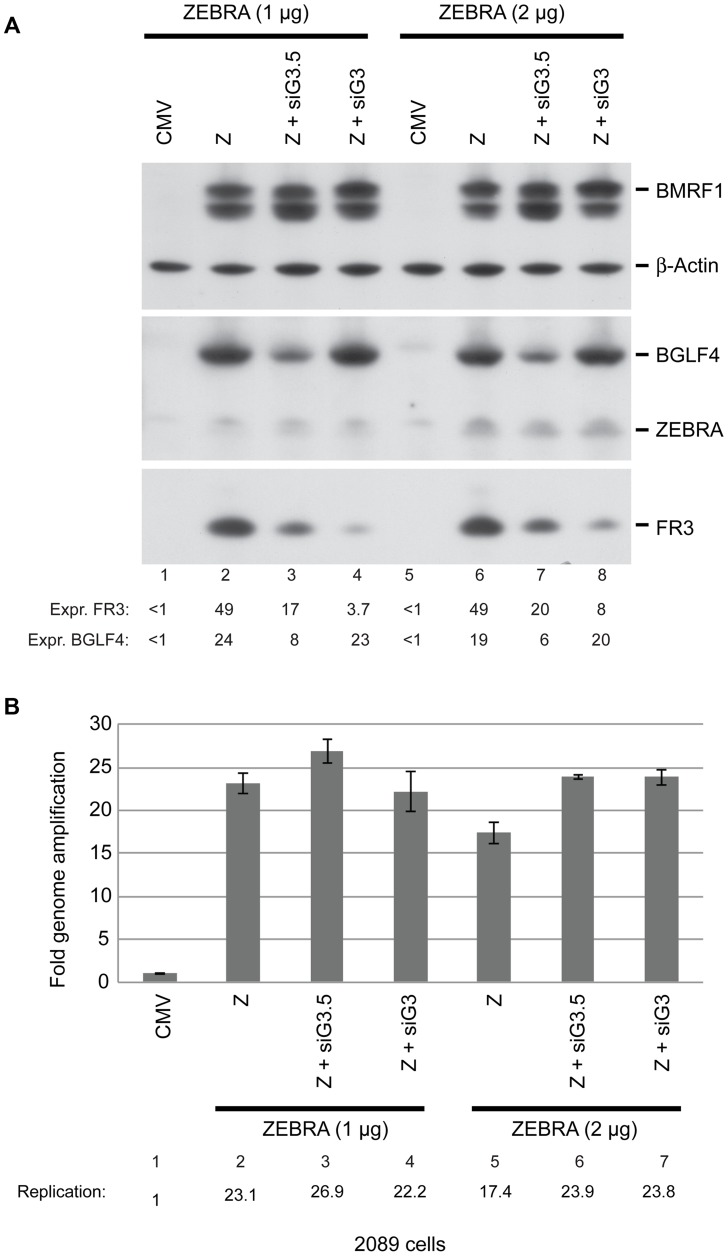
BGLF3 regulates late gene expression. A) Western blot analysis of EBV lytic proteins to study the effect of knocking down expression of BGLF3 and BGLF3.5. 2089 cells were transfected with two concentrations of the ZEBRA expression plasmid and siRNAs to BGLF3 (siG3) and BGLF3.5 (siG3.5). Cells were harvested after 48 hours of transfection. The membrane was blotted with antibodies specific to BGLF4, ZEBRA and the late FR3 protein. The level of β-actin used as a loading control. B) Total DNA was prepared from the same samples and was analyzed by quantitative PCR for the level of the upstream region of oriLyt as a marker for EBV replication. The relative DNA concentration was calculated using the standard curve method. The experiment is a representation of two biological replicates.

To investigate the possibility that BGLF4 might exert its effects on late gene expression by controlling expression of a second tier of late gene regulators we studied the effect of siRNA to BGLF3 and BGLF3.5 on synthesis of late products. 2089 cells transfected with two different concentrations of ZEBRA expression vector (1 and 2 µg DNA) in the presence of siRNA were harvested after 48 h. Cell lysates were analyzed using Western blot assays. The experiment illustrated in figure 9A is a representative of two biological replicates. Knockdown of BGLF3 significantly reduced expression of the late FR3 protein by an average of 9.5-fold, but siRNA to BGLF3 (siG3) had no effect on expression of ZEBRA, BGLF4, or EA-D (Fig. 8B and 9A). Silencing of BGLF3.5 reduced FR3 expression by an average of 2.7-fold. However, siRNA to BGLF3.5 (siG3.5) slightly reduced expression of BGLF4 but not ZEBRA or EA-D. To assess the effect of both siRNAs on viral DNA replication we purified DNA from the same cells and examined the level of viral DNA using qPCR. Neither of the two siRNAs reduced the level of viral DNA in cells expressing ZEBRA (Fig. 9B). We used RT-qPCR to demonstrate that the siRNA to BGLF3 reduced the level of the endogenous BGLF3 transcript by 2.7-fold, but had no effect on the level of BMRF1 mRNA (Fig. S5). Expression of four other late transcripts, BDLF1, BdRF1, BLLF1, and BcLF1, was reduced as a result of silencing BGLF3 (Fig. S6). These results demonstrate that BGLF3 is necessary for progress of the EBV lytic cycle into the late phase.

### siG3-resistant BGLF3 (rG3) rescued expression of late genes

**Figure 10:**
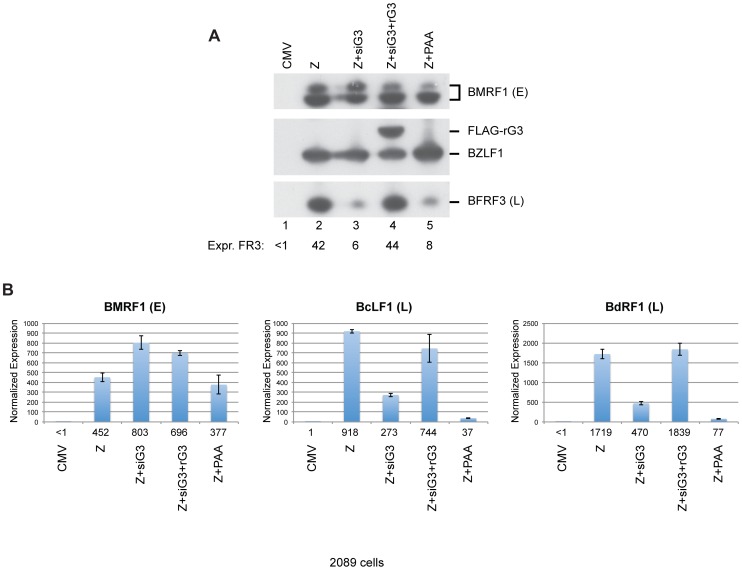
Silent mutations in BGLF3 suppress the effect of siG3 on late gene expression. (A) Western blot analysis of 2089 cells transfected with the indicated molecules. The membrane was blotted with specific antibodies against three EBV lytic proteins: ZEBRA, BMRF1 (E: early), and BFRF3 (L: late). FLAG antibody was used to detect the siG3-resistant BGLF3 (rG3). (B) RT-qPCR to assess the capacity of rG4 to rescue expression of two late transcripts, BcLF1 and BdRF1. PAA, phosphonoacetic acid, an inhibitor of viral DNA replication.

To demonstrate that the effect of siG3 on late gene expression was specific to knockdown of the *bglf3* gene, we inserted silent mutations in the region of the BGLF3 mRNA that is recognized by siG3. These mutations disrupt the complementarity between siG3 and the BGLF3 transcript without altering the amino acid sequence of the protein. Insertion of these mutations resulted in a form of BGLF3, referred to as rG3, which is resistant to siG3. Co-transfection of ZEBRA plus siG3 in 2089 cells reduced the level of FR3 by 7-fold relative to cells solely transfected with ZEBRA (Fig. 10A, lanes 2 and 3). Ectopic expression of rG3 in cells transfected with ZEBRA and siG3 restored expression of the late FR3 protein to a level equivalent to that observed in cells expressing ZEBRA alone (Fig. 10A, compare lanes 2 and 4). The experiment was repeated twice and similar results were obtained. To assess the effect of rG3 on expression of other late genes, we purified total RNA from the same set of samples and examined expression of BMRF1, an early gene, and two late genes: BcLF1, and BdRF1. Using RT-qPCR, we found that siG3 reduced the level of the two late transcripts but had no effect on the level of the early BMRF1 transcript; co-transfection of rG3 rescued expression of BcLF1 and BdRF1 (Fig. 10B). These results demonstrate that BGLF3 is indispensable for expression of late genes.

### Both BGLF4 and BGLF3 are indispensable for late gene expression

To determine whether BGLF3 or BGLF3.5 can substitute for the role of BGLF4 in activation of late gene expression, we knocked down BGLF4 by transfecting siG4 and expressed BGLF3 and BGLF3.5, separately and together, using expression vectors. We found that provision of BGLF3 and BGLF3.5 was not sufficient to trigger expression of the late FR3 protein in absence of BGLF4 (Fig. S7). Similarly, ectopic expression of BGLF3 and BGLF3.5 did not restore late gene expression in cells treated with phosphonoacetic acid (PAA), an inhibitor of viral DNA replication (Fig. S7, lanes 8 and 9). These results demonstrate that BGLF4, BGLF3 and viral DNA replication are three necessary components for progress of the EBV lytic cycle into the late phase.

## Discussion

**Figure 11:**
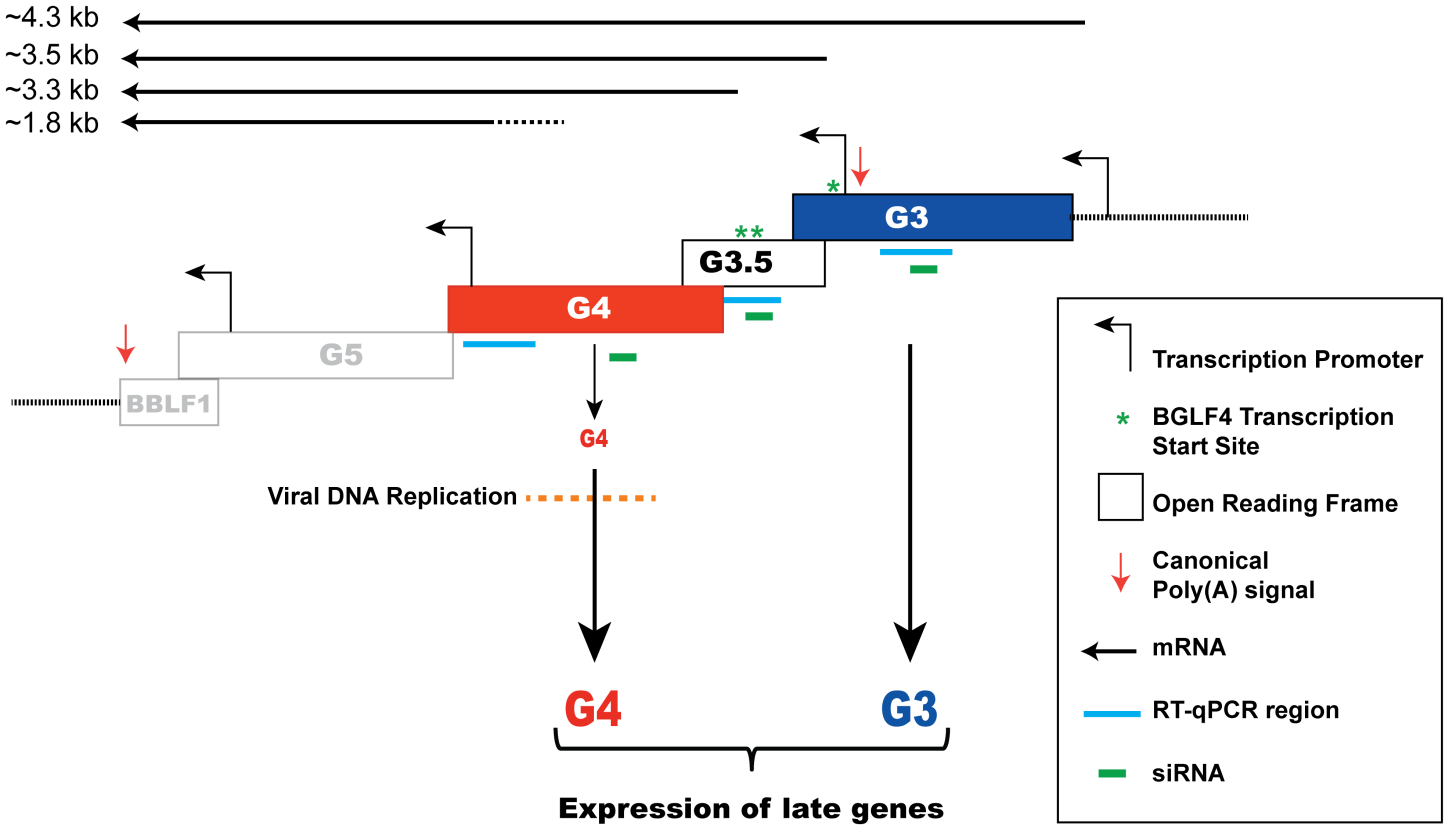
A model depicting regulation of late gene expression by the G-locus. A schematic diagram illustrating the organization of the *bglf4* (G4), *bglf3.5* (G3.5) and *bglf3* (G3) open reading frames in the EBV genome. Four canonical TATA box elements located in this locus are present upstream of the *bglf3*, *bglf3.5*, *bglf5* and *bblf1* genes [33], [47], [73]. The asterisks denote three mapped transcription start sites of transcripts encoding BGLF4. A poly(A) signal is located at the 3′ end of the *bblf1* gene. Solid lines represent the predicted transcripts synthesized from this locus. Our findings postulate that both BGLF4 and BGLF3 function to activate expression of late genes.

Classification of lytic viral gene expression into pre- and post-replication temporal phases is applicable to the life cycles of all herpesviruses. While kinetics of expression of pre-replication genes has been extensively studied, little is known about the order of events that take place prior to expression of late genes. Our findings reveal a previously unknown transitional step between EB viral DNA replication and late gene expression. We demonstrate that the kinase function of BGLF4 is necessary for optimal expression of late genes. Inactivating the kinase activity of BGLF4 markedly impairs late gene expression without impeding viral DNA replication; the kinase dead mutant supported viral DNA replication to the same extent as wild-type BGLF4 (Fig. S2, 2, and 5). Disrupting the kinase activity or the expression of BGLF4 selectively reduced the level of late transcripts (Fig. 3, 6 and 7). Analysis of the EBV transcriptome revealed that expression of 31 late genes was reduced in the absence of BGLF4 (Fig. 7). Silencing of BGLF4 did not reduce the level of most early transcripts including those encoding replication proteins. Using siRNA to compare the expression pattern of EBV genes with and without BGLF4 we identified two non-late genes, *bglf3* and *bglf3.5*, whose expression was significantly reduced as a result of silencing BGLF4 (Fig. 7, 8 and S3). By knockdown experiments, we found that BGLF3 is an independent regulator of late gene expression. siRNA to BGLF3 markedly reduced the level of FR3, a canonical late protein, and the transcripts of several other late genes without affecting endogenous expression of BGLF4 or viral DNA replication (Fig. 8B, 9A, 10 and S6). Expression of siRNA resistant forms of BGLF3 and BGLF4 provided evidence that each protein plays an independent and indispensable role in regulation of late gene expression (Fig. 5, 6, and 10). In summary, our results identify a control locus composed of BGLF4 and BGLF3 that regulates synthesis of EBV encoded structural proteins (Fig. 11).

### Role of BGLF4 in regulating viral DNA replication and late gene expression

To establish a distinct regulatory role of BGLF4 in activation of late gene expression it is imperative to exclude that this role results from an effect on viral DNA replication. Several groups have assessed the effect of knocking down or knocking out BGLF4 on the process of EBV genome amplification. Two separate research groups reported that knocking out the *bglf4* gene did not affect the level of viral DNA synthesized during the lytic cycle [28], [32]; three other groups found that lack of BGLF4 reduced viral DNA replication by 1.4- to 2-fold [31], [44], [47]. We found that ectopic expression of BGLF4 in BGLF4 null cells enhanced viral DNA replication by 1.6 to 2.6-fold (Fig. 1, S2, and 2) in agreement with the second group of studies. These slight differences in the effect of BGLF4 on viral DNA replication could be attributed to the cell background or the approach used to knockout the *bglf4* gene. In our knockdown experiments we found that silencing of BGLF4 in BZKO cells reduced viral DNA replication by 2-fold and in 2089 cells by 1.1-fold (Fig. 4 and 5). The effect of BGLF4 on viral DNA replication was independent of its kinase activity. In delta G4/G5 cells, expression of either wild-type BGLF4 or the kinase inactive mutant enhanced replication to same extent (Fig. 2 and S2). Thus, we conclude that the kinase activity of BGLF4 is required for its effect on late gene expression but not for its modest enhancement of viral DNA replication.

Since expression of late genes is stipulated by the onset of viral genome amplification we sought to segregate the effect of BGLF4 on replication from its effect on expression of late genes. We achieved this goal using two different approaches: first, we varied the concentration of siRNA to BGLF4. We demonstrated that at a very low concentration of BGLF4 protein there was significant reduction in late gene expression without any significant compromise in viral DNA replication (Fig. 4, compare lane 7 with lane 8). As we increased the concentration of BGLF4, viral DNA replication remained unchanged (Fig. 4B) while late gene expression increased proportionally (Fig. 4A). Second, we supplied a kinase dead form of BGLF4 to cells with a knockout of the *bglf4* gene. Viral DNA replication was rescued to normal levels but there remained a marked defect in late gene expression (Fig. S2 and 2).

The possibility that BGLF4 possesses additional functions that are independent of its kinase activity is supported by the capacity of ORF36, a BGLF4 homolog encoded by MHV68, to repress the function of class 1 and 2 histone deacetylases (HDACs) [43]. Repression of HDAC1 and 2 by ORF36 is mediated by protein-protein interactions, rather than by phosphorylation. Expression of either wild-type or kinase inactive ORF36 enhances MHV68 early gene expression and viral DNA replication [43]. The effect of BGLF4 or BGLF4(K102I) on EB viral DNA replication may also be due to the capacity to interact with and disrupt the function of HDAC1 and 2 during the early phase of the lytic cycle. BGLF4 interacts with HDAC1 and 2 [43]; the biological significance of such interaction for temporal control of the EBV life cycle has not been studied.

### Expression of BGLF4, BGLF3.5 and BGLF3 from the late control locus in the EBV genome

Expression profiling of EBV genes during lytic infection affirms that most late genes are subject to regulation by BGLF4 (Fig. 7). By comparing the EBV transcriptome during lytic infection in the absence and presence of BGLF4 we found that BGLF3 and BGLF3.5 represent the two most down-regulated non-late genes in cells transfected with siG4 (Fig. 7). The effect of siG4 on the level of BGLF3 and BGLF3.5 mRNAs could be attributed to two different mechanisms. First, in addition to targeting the BGLF4 mRNA, siG4 might concurrently knock down the transcripts encoding BGLF3 and BGLF3.5. *bglf3*, *bglf3.5* and *bglf4* are nested within a transcription unit containing five overlapping open reading frames that also includes *bglf5* and *bblf1*, respectively (Fig. 11). Due to the presence of a single canonical poly(A) signal at the 3′ end of *bblf1*, transcription from this locus is likely to result in co-terminal transcripts (Fig. 8D) [33], [62], unless cryptic variants of the poly(A) signal exist at the 3′end of some genes [63]. In figure 8A, knockdown of BGLF4 abolished all the RNA species that were predicted to contain BGLF4 sequence. This result is in favor of co-terminal expression of transcripts encoded by the late control locus. Second, BGLF4 might regulate the level of the BGLF3 and BGLF3.5 mRNAs. In Fig. S4, siG4 down-regulated the level of BGLF3 mRNA; provision of rG4 overcame the effect of siG4 and increased the level of the endogenous BGLF3 transcript by 4-fold. BGLF4 might either enhance the activity of the BGLF3 promoter or enhance the stability of the BGLF3 mRNA.

Silencing of BGLF3 had no discernable effect on the protein level of BGLF4 (Fig. 8B and 9A). A possible explanation of this result is that BGLF3 is expressed at a low level during the late phase of the lytic cycle. Northern blot analysis revealed that the 4.3 kb transcript, the only RNA containing the BGLF3 sequence, was considerably less abundant relative to the other RNA species synthesized from this locus (Fig. 8A and C). Therefore, knockdown of the 4.3 kb RNA (Fig. 8D, transcript A) presumably had more impact on the level of BGLF3 relative to the overall level of BGLF4 containing mRNAs (Fig. 8C and D). In two independent experiments, abolishing the BGLF3 containing transcript by siG3 reduced the abundance of the FR3 protein without affecting the level of the BGLF4 protein (Fig. 8B and 9A).

Contrary to siG3, siRNA to BGLF3.5 (siG3.5) reduced the level of the BGLF4 protein by 3-fold (Fig. 9A, lanes 3 and 7). BGLF4 has three transcriptional start sites; two sites mapped to regions within the *bglf3.5* coding sequence and a third site mapped upstream of the translation start codon of *bglf3.5*. Reduction in the level of BGLF4 protein by siG3.5 is likely due to the ability of siG3.5 to target transcripts that encode both BGLF3.5 and BGLF4 (Fig. 11). In KSHV, ORF36, the homolog of BGLF4, is robustly translated as a downstream cistron from a polycistronic transcript that initiates with ORF35, the KSHV homolog of BGLF3.5 [64], [65].

### Both BGLF4 and BGLF3 are independently necessary for late gene expression

The kinase activity of BGLF4 and not the mere synthesis of the BGLF4 transcript or protein is necessary for stimulation of late gene expression (Fig. 2, S2, 3, 5, and 6). In knockout and knockdown experiments of BGLF4, expression of the kinase active, and not the kinase dead, form of BGLF4 activated synthesis of late transcripts. Attempts to complement the lack of BGLF4 with expression of BGLF3, BGLF3.5 or both did not restore expression of late genes (Fig. S7). Therefore, the role of BGLF4 in late gene expression is not limited to regulating the level of BGLF3; BGLF4 has an unknown but indispensable function in the mechanism regulating synthesis of late products. BGLF3 is an ortholog of the MHV68-encoded ORF34 [4]. Mutant MHV68 virus that lacks the *orf34* gene underwent viral genome amplification but failed to express late products. Here, we found that the function of MHV-68 ORF34 in inducing late gene expression is conserved in EBV BGLF3. Silencing expression of BGLF3 using siRNAs abolished synthesis of the FR3 late protein and the transcripts of several late genes without affecting the level of BGLF4 or viral DNA replication (Fig. 8, 9, and S6). Providing a form of BGLF3 that is resistant to siG3 annulled the effect of the siRNA on expression of late genes. The exact role of BGLF3 in regulation of EBV late gene expression has not been established. Knockout of ORF34, the murine homolog of BGLF3, abolished recruitment of RNAPII to late promoters. In a related observation, we found that BGLF3 interacts with the C-terminal domain of RPB1, the large catalytic subunit of RNAPII (data not shown). These results represent the first demonstration of the indispensable roles of BGLF4 and BGLF3 in regulation of EBV late gene expression. However, ectopic expression of BGLF4 or BGLF3 does not complement the lack of viral DNA replication (Fig. S7, lanes 8 and 9, and data not shown). Therefore, viral DNA replication as well as the function of BGLF4 and BGLF3 represent three essential components for stimulation of late gene expression. Our findings have scientific and translational implications. The findings provide new insight on regulation of late gene expression, one of the main puzzles in virology, and emphasize the importance of targeting the kinase activity of BGLF4 for development of new antiviral drugs.

## Materials and Methods

### Expression vectors

The vector encoding the ZEBRA protein was prepared as previously described [66]. Constructs expressing wild-type BGLF4 and the kinase dead BGLF4(K102I) were a kind gift of Dr. Mei-Ru Chen [16]. BGLF4 forms that are resistant to the corresponding siRNA (siG4) were generated by introducing silent point mutations using the following mutagenic primer: 5′-GTGACCAACATTGATGACATGACGGAGACATTATACGTCAAATTACCTGAAAACATGACGCGCTGTGATCACCTCCCCATTACC-3′ and its complementary strand: 5′-GGTAATGGGGAGGTGATCACAGCGCGTCATGTTTTCAGGTAATTTGACGTATAATGTCTCCGTCATGTCATCAATGTTGGTCAC-3′.

### Cell culture and transfection

2089, Bam Z knockout (BZKO), and delta BGLF4/BGLF5 (delta G4/G5) are 293 human embryonic kidney (HEK) cells stably transfected with bacmids containing wild-type, BZLF1 null, and BGLF4/BGLF5 null EBV B95.8 genomes, respectively [47], [67], [68]. Mutations in wild type EBV bacmid (2089) were generated by homologous recombination in which the *bzlf1* gene and the *bglf4* gene were replaced with the kanamycin resistance gene [47], [67]. Disruption of the *bglf4* gene abolished expression of BGLF4 and BGLF5. The cells were cultured in Dulbecco's modified Eagle medium (DMEM) supplemented with 10% fetal bovine serum (FBS), and antibiotics (penicillin-streptomycin at 50 units/ml and amphotericin B at 1 µg/ml). Hygromycin B (Calbiochem) 100 µg/ml was added to the medium to select for 293 cells containing wild type or mutant EBV bacmids. Transfection of eukaryotic plasmids was performed in 25 cm^2^ flasks using 36 µl DMRIE-C transfection reagent (Invitrogen) mixed with 2 µg of ZEBRA expression vector to induce the lytic cycle, and 3 µg of plasmids encoding wild-type or kinase dead BGLF4.

Silencing expression of BGLF4, BGLF3 and BGLF3.5 was attained using RNA interference technique. Transfections were performed using Lipofectamine 2000 (Invitrogen) following the manufacturer's protocol. Several siRNAs were tested to knockdown BGLF4, BGLF3, and BGLF3.5. The most specific siRNA was then used in knockdown experiments to study the role of each of these proteins during the lytic cycle. All transfections were carried out in OPTI-MEM medium. Cells were incubated at 37°C in 5% CO_2_ incubator and harvested after 48 h of transfection.

### Protein detection

Harvested cells were re-suspended in sodium dodecyl sulfate (SDS) sample buffer at 10^6^ cells/10 µl. Proteins were separated in 10% SDS-polyacrylamide gels and transferred to nitrocellulose membranes (Bio-Rad). The membrane was blotted with specific antibodies to cellular and viral proteins. The BGLF4 antibody was raised against amino acids 1 to 220 in rabbit. BGLF4(1–220) was expressed in *Escherichia coli* from a pET-22b vector and purified using nickel affinity chromatography. The EA-D (BMRF1) monoclonal antibody (R3.1) was kindly provided by G. Pearson [69]. Anti-ZEBRA and anti-BFRF3 are polyclonal rabbit antibodies and were described previously [54]. FLAG-tagged proteins were detected with anti-FLAG mouse monoclonal antibody (Sigma). β-actin was detected using a mouse monoclonal antibody (Sigma). The antigen-antibody complex was detected by autoradiography using ^125^I-protein A.

### Quantitative RT-PCR

RNA was prepared from cells harvested 48 h after transfection using Qia-shredder and RNeasy Plus products from Qiagen. The concentration of RNA in each sample was determined by measuring the optical density at 260 nm. The level of viral transcripts was assessed in 100 ng of total RNA using iScript One-Step RT-PCR with SYBR green (Bio-Rad) in a total volume of 25 µl. The level of 18S rRNA was measured to normalize for the total amount of RNA. Each sample was analyzed in triplicates and the fold change in expression was calculated using the ΔΔC_T_ formula. The sequences of the primers used are available upon request.

### Next generation RNA sequencing and data analysis

Three strand-specific sequencing libraries were produced from total RNA of 2089 cells transfected with empty vector, ZEBRA or ZEBRA plus siG4 as previously described [70]. The libraries were run on HiSeq 2000, generating approximately 86, 47 and 49 million reads, respectively. The generated reads were single-end and each read was 76 bp long. Adapter sequences, empty reads, and low-quality sequences were removed. Also, the first 16 and last 4 nucleotides in each read were trimmed using the FASTX toolkit (http://hannonlab.cshl.edu/fastx_toolkit/index.html) to remove low quality bases. Trimmed reads were mapped to the human reference genome (hg19) with a known transcriptome index (UCSC Known Gene annotation) using Tophat v2.0.8 [71]. Those reads that did not map to the human genome were later mapped to the EBV genome (GenBank accession number NC_007605.1) with a known transcriptome annotation [62]. We used the Expectation-Maximization (EM) algorithm in RSEM [57] with Bowtie 2 [72] to estimate gene expression levels. EBSeq within RSEM pipeline was used to identify differentially expressed genes [56].

### Replication assay

Cells were harvested and re-suspended in 400 µl of lysis buffer containing 50 mM Tris-HCl [pH 8.1], 1% SDS, and 10 mM EDTA. The cell lysate was sonicated twice, 10 pulses each, and centrifuged for 10 min in a microfuge at full speed. The supernatant was diluted 10-fold in dilution buffer containing 16.7 mM Tris-HCl [pH 8.1], 0.01% SDS, 1.1% Triton X-100, 167 mM NaCl, and 1.2 mM EDTA. The samples were subjected to Proteinase-K digestion (Roche) followed by phenol-chloroform extraction to remove proteins. The DNA was purified using Qiagen PCR-purification kit. The total DNA content was determined by measuring the absorbance at 260 nm. The extent of viral genome amplification was quantitated using the IQ Sybr Green SuperMix kit (Bio-Rad). The sequences of the forward and reverse oriLyt primers are: 5′-TCCTCTTTTTGGGGTCTCTG-3′ and 5′-CCCTCCTCCTCTCGTTATCC-3′. The relative concentration of DNA was calculated based on a standard curve constructed from different concentrations of oriLyt. The level of viral DNA was normalized to control sample transfected with empty vector (CMV).

#### Northern blot

Cell pellets were collected at 48 h after transfection with CMV, ZEBRA, and siRNAs. Total RNA was isolated using the RNeasy Plus kit (QIAGEN) and the resulting RNA was quantified via UV spectrometry. Thirty micrograms of RNA per sample was electrophoresed on a 1% agarose-6% formaldehyde gel in 20 mM MOPS (morpholinepropanesulfonic acid; pH 7). The gel was transferred to a nylon membrane (HYBOND-N+; Amersham Biosciences) and hybridized with ^32^P-labeled probes. The radioactive probes were complementary to unique sequences in BGLF4, BGLF3.5, and RNaseP HI. Probes were prepared by random priming. The BGLF4 probe was generated from a 620-bp PCR product amplified using the following primers: 5′-ATGGATGTGAATATGGCTGC-3′ and 5′-TCTGTGAAATCCACCAGGAT-3′. The BGLF3.5 probe is a PCR fragment that was amplified using the following primers: 5′- TAGAAAAGAGAGCGGCTGTG-3′ and 5′-CCGCTCTAGCTGCTTGTAGT-3′. The RNaseP HI probe was prepared using a 350-bp NcoI-to-PstI fragment of cDNA from the HI RNA component of human RNaseP.

## Supporting Information

Figure S1 **BGLF4 induces expression of the late FR3 protein.** In Fig. 1A, we showed that expression of BGLF4, but not BGLF5, in delta G4/G5 cells up-regulated expression of FR3. To confirm this result we repeated the experiment in the same cell line and found that BGLF4 is necessary for efficient expression of the late FR3 protein. Complementation with BGLF5 did not up-regulate the level of FR3 protein (lane 4).
(TIF)

Figure S2 **The kinase activity of BGLF4 is necessary for its role in activating late gene expression.** (A) Expression of viral lytic proteins in delta G4/G5 cells transfected with empty vector (CMV), ZEBRA (Z), BGLF4, ZEBRA plus BGLF4, kinase dead BGLF4(K102I), and ZEBRA plus BGLF4(K102I). The blot was sequentially reacted with antibodies specific to BFRF3, ZEBRA, BGLF4, and BMRF1. Since BGLF4 and BMRF1 cannot be fully resolved on 10% SDS-PAGE, figure 2A has two panels displaying the BGLF4 protein with and without BMRF1. (B) Fold change in the level of EBV DNA was determined by quantitative PCR in delta G4/G5 cells transfected with the indicated expression vectors relative to empty vector.
(TIF)

Figure S3 **Knockdown of BGLF4 down-regulates expression of BGLF3 and BGLF3.5.** Quantitative RT-PCR was used to measure the level of the BGLF3 (A) and BGLF3.5 (B) transcripts in 2089 cells with and without silencing expression of BGLF4. Total cellular RNA was harvested at 48 h after transfection. The relative level of each transcript was calculated as a fold-change relative to empty vector using the ΔΔ*C_T_* method.
(TIF)

Figure S4 **siG4-resistant BGLF4 (rG4) enhances expression of BGLF3.** Knockdown of BGLF4 markedly reduced the level of the BGLF3 transcript. Two possible interpretations could be envisioned: i) BGLF4 regulates expression of BGLF3 and ii) BGLF4 and BGLF3 are encoded by the same transcript. To determine if ectopic expression of BGLF4 can up-regulate the level of the BGLF3 mRNA transcribed from the endogenous viral genome, we knocked down the endogenous BGLF4 transcript using siG4 and co-transfected a form of BGLF4 that is resistant to the siRNA. Using RT-qPCR we found that silencing of BGLF4 reduced the level of BGLF3 by 2.8-fold relative to cells expressing ZEBRA. Co-expression of rG4 enhanced expression of BGLF3 by 4-fold compared to cells transfected with ZEBRA plus siG4. This result indicates that BGLF4 has the capacity to induce expression of the endogenous BGLF3 transcript.
(TIF)

Figure S5 **siG3 specifically reduces the level of the BGLF3 transcript.** In Fig. 9, we found that siG3 reduced expression of the FR3 late protein without affecting viral DNA replication. To determine if the effect of siRNA to BGLF3 is due to its capacity to target the BGLF3 transcript, we prepared total RNA from samples used in figure 9. Using RT-qPCR we assessed the level of BGLF3 and BMRF1 mRNAs in 2089 cells transfected with wild-type ZEBRA in the absence and presence of siG3. Expression of ZEBRA (Z) stimulated the BMRF1 transcript by 887-fold and the BGLF3 transcript by 41-fold relative to cells transfected with empty vector (CMV). Co-transfection of siG3 had no effect on the level of the BMRF1 transcript but markedly reduced the level of BGLF3 mRNA by 2.7-fold. These results together with data in Fig. 9 demonstrate that the observed effect of siG3 is due to reduction in amount of the BGLF3 transcript.
(TIF)

Figure S6 **BGLF3 is necessary for expression of late genes.** To investigate the effect of silencing BGLF3 on expression of late genes other than *bfrf3* (Fig. 9A), we used RT-qPCR to assess the level of four late transcripts, BDLF1, BdRF1, BLLF1, and BcLF1, in the same RNA samples studied in Fig. S5. We found that knockdown of BGLF3 reduced the level of these late transcripts in 2089 cells transfected with ZEBRA plus siG3 relative to cells expressing ZEBRA alone by 11.2, 9.1, 10.1, and 5.9-fold respectively. In the same experiment (Fig. 9), the level of BGLF4 protein was not affected by siG3. These results demonstrate that BGLF3 plays an essential role in regulating expression of late genes during the EBV lytic cycle.
(TIF)

Figure S7 **Expression of BGLF3 and BGLF3.5 is not sufficient to activate late gene expression in the absence of BGLF4.** We examined whether compensatory expression of BGLF3 and BGLF3.5 in the absence of BGLF4 would rescue expression of the late FR3 protein. 2089 cells were transfected with the indicated expression vectors with and without siG4. Cells were harvested after 48 h and expression of lytic proteins was analyzed by Western blot. We found that knockdown of BGLF4 abolished synthesis of FR3 (compare lane 2 with lane 4). Ectopic expression of BGLF3 and BGLF3.5 in 2089 cells with siG4 failed to restore expression of the late FR3 protein (compare lane 3 with lane 7). This finding indicates that BGLF3 and BGLF3.5 do not substitute for the role of BGLF4 in regulation of late gene expression; BGLF4 has an independent role in activation of late gene expression.
(TIF)

## References

[1] Song MJ, Hwang S, Wong WH, Wu TT, Lee S, et al (2005) Identification of viral genes essential for replication of murine gamma-herpesvirus 68 using signature-tagged mutagenesis. Proceedings of the National Academy of Sciences of the United States of America 102: 3805–3810. 15738413 10.1073/pnas.0404521102 PMC553290

[2] Arumugaswami V, Wu TT, Martinez-Guzman D, Jia Q, Deng H, et al (2006) ORF18 is a transfactor that is essential for late gene transcription of a gammaherpesvirus. J Virol 80: 9730–9740. 16973577 10.1128/JVI.00246-06 PMC1617240

[3] Wong E, Wu TT, Reyes N, Deng H, Sun R (2007) Murine gammaherpesvirus 68 open reading frame 24 is required for late gene expression after DNA replication. J Virol 81: 6761–6764. 17392360 10.1128/JVI.02726-06 PMC1900117

[4] Wu TT, Park T, Kim H, Tran T, Tong L, et al (2009) ORF30 and ORF34 are essential for expression of late genes in murine gammaherpesvirus 68. J Virol 83: 2265–2273. 19091863 10.1128/JVI.01785-08 PMC2643722

[5] Perng YC, Qian Z, Fehr AR, Xuan B, Yu D (2011) The human cytomegalovirus gene UL79 is required for the accumulation of late viral transcripts. Journal of virology 85: 4841–4852. 21367901 10.1128/JVI.02344-10 PMC3126216

[6] Isomura H, Stinski MF, Murata T, Yamashita Y, Kanda T, et al (2011) The human cytomegalovirus gene products essential for late viral gene expression assemble into prereplication complexes before viral DNA replication. Journal of virology 85: 6629–6644. 21507978 10.1128/JVI.00384-11 PMC3126524

[7] Wyrwicz LS, Rychlewski L (2007) Identification of Herpes TATT-binding protein. Antiviral research 75: 167–172. 17400302 10.1016/j.antiviral.2007.03.002

[8] Gruffat H, Kadjouf F, Mariame B, Manet E (2012) The Epstein-Barr virus BcRF1 gene product is a TBP-like protein with an essential role in late gene expression. Journal of virology 86: 6023–6032. 22457524 10.1128/JVI.00159-12 PMC3372218

[9] Serio TR, Cahill N, Prout ME, Miller G (1998) A functionally distinct TATA box required for late progression through the Epstein-Barr virus life cycle. Journal of Virology 72: 8338–8343. 9733880 10.1128/jvi.72.10.8338-8343.1998 PMC110205

[10] Isomura H, Stinski MF, Kudoh A, Murata T, Nakayama S, et al (2008) Noncanonical TATA sequence in the UL44 late promoter of human cytomegalovirus is required for the accumulation of late viral transcripts. Journal of virology 82: 1638–1646. 18057245 10.1128/JVI.01917-07 PMC2258720

[11] Tang S, Yamanegi K, Zheng ZM (2004) Requirement of a 12-base-pair TATT-containing sequence and viral lytic DNA replication in activation of the Kaposi's sarcoma-associated herpesvirus K8.1 late promoter. J Virol 78: 2609–2614. 14963167 10.1128/JVI.78.5.2609-2614.2004 PMC369211

[12] Heilmann AM, Calderwood MA, Portal D, Lu Y, Johannsen E (2012) Genome-wide analysis of epstein-barr virus rta DNA binding. Journal of virology 86: 5151–5164. 22379087 10.1128/JVI.06760-11 PMC3347379

[13] Chua HH, Lee HH, Chang SS, Lu CC, Yeh TH, et al (2007) Role of the TSG101 gene in Epstein-Barr virus late gene transcription. J Virol 81: 2459–2471. 17182691 10.1128/JVI.02289-06 PMC1865947

[14] Wing BA, Johnson RA, Huang ES (1998) Identification of positive and negative regulatory regions involved in regulating expression of the human cytomegalovirus UL94 late promoter: role of IE2-86 and cellular p53 in mediating negative regulatory function. J Virol 72: 1814–1825. 9499032 10.1128/jvi.72.3.1814-1825.1998 PMC109471

[15] DeMeritt IB, Podduturi JP, Tilley AM, Nogalski MT, Yurochko AD (2006) Prolonged activation of NF-kappaB by human cytomegalovirus promotes efficient viral replication and late gene expression. Virology 346: 15–31. 16303162 10.1016/j.virol.2005.09.065 PMC2600890

[16] Chen MR, Chang SJ, Huang H, Chen JY (2000) A protein kinase activity associated with Epstein-Barr virus BGLF4 phosphorylates the viral early antigen EA-D in vitro. J Virol 74: 3093–3104. 10708424 10.1128/jvi.74.7.3093-3104.2000 PMC111808

[17] Gershburg E, Pagano JS (2008) Conserved herpesvirus protein kinases. Biochim Biophys Acta 1784: 203–212. 17881303 10.1016/j.bbapap.2007.08.009 PMC2265104

[18] Romaker D, Schregel V, Maurer K, Auerochs S, Marzi A, et al (2006) Analysis of the structure-activity relationship of four herpesviral UL97 subfamily protein kinases reveals partial but not full functional conservation. J Med Chem 49: 7044–7053. 17125257 10.1021/jm060696s

[19] Kuny CV, Chinchilla K, Culbertson MR, Kalejta RF (2010) Cyclin-dependent kinase-like function is shared by the beta- and gamma- subset of the conserved herpesvirus protein kinases. PLoS pathogens 6: e1001092. 20838604 10.1371/journal.ppat.1001092 PMC2936540

[20] Kawaguchi Y, Kato K, Tanaka M, Kanamori M, Nishiyama Y, et al (2003) Conserved protein kinases encoded by herpesviruses and cellular protein kinase cdc2 target the same phosphorylation site in eukaryotic elongation factor 1delta. J Virol 77: 2359–2368. 12551973 10.1128/JVI.77.4.2359-2368.2003 PMC141098

[21] Lee CP, Chen JY, Wang JT, Kimura K, Takemoto A, et al (2007) Epstein-Barr virus BGLF4 kinase induces premature chromosome condensation through activation of condensin and topoisomerase II. J Virol 81: 5166–5180. 17360754 10.1128/JVI.00120-07 PMC1900198

[22] Chen PW, Lin SJ, Tsai SC, Lin JH, Chen MR, et al (2010) Regulation of microtubule dynamics through phosphorylation on stathmin by Epstein-Barr virus kinase BGLF4. J Biol Chem 285: 10053–10063. 20110360 10.1074/jbc.M109.044420 PMC2843168

[23] Zhu J, Liao G, Shan L, Zhang J, Chen MR, et al (2009) Protein array identification of substrates of the Epstein-Barr virus protein kinase BGLF4. J Virol 83: 5219–5231. 19244323 10.1128/JVI.02378-08 PMC2682057

[24] Iwahori S, Murata T, Kudoh A, Sato Y, Nakayama S, et al (2009) Phosphorylation of p27Kip1 by Epstein-Barr virus protein kinase induces its degradation through SCFSkp2 ubiquitin ligase actions during viral lytic replication. J Biol Chem 284: 18923–18931. 19451650 10.1074/jbc.M109.015123 PMC2707218

[25] Kudoh A, Daikoku T, Ishimi Y, Kawaguchi Y, Shirata N, et al (2006) Phosphorylation of MCM4 at sites inactivating DNA helicase activity of the MCM4-MCM6-MCM7 complex during Epstein-Barr virus productive replication. Journal of virology 80: 10064–10072. 17005684 10.1128/JVI.00678-06 PMC1617282

[26] Kato K, Kawaguchi Y, Tanaka M, Igarashi M, Yokoyama A, et al (2001) Epstein-Barr virus-encoded protein kinase BGLF4 mediates hyperphosphorylation of cellular elongation factor 1delta (EF-1delta): EF-1delta is universally modified by conserved protein kinases of herpesviruses in mammalian cells. J Gen Virol 82: 1457–1463. 11369891 10.1099/0022-1317-82-6-1457

[27] Lee CP, Huang YH, Lin SF, Chang Y, Chang YH, et al (2008) Epstein-Barr virus BGLF4 kinase induces disassembly of the nuclear lamina to facilitate virion production. J Virol 82: 11913–11926. 18815303 10.1128/JVI.01100-08 PMC2583647

[28] Meng Q, Hagemeier SR, Kuny CV, Kalejta RF, Kenney SC (2010) Simian virus 40 T/t antigens and lamin A/C small interfering RNA rescue the phenotype of an Epstein-Barr virus protein kinase (BGLF4) mutant. J Virol 84: 4524–4533. 20147387 10.1128/JVI.02456-09 PMC2863785

[29] Sun X, Bristol JA, Iwahori S, Hagemeier SR, Meng Q, et al (2013) Hsp90 inhibitor 17-DMAG decreases expression of conserved herpesvirus protein kinases and reduces virus production in Epstein-Barr virus-infected cells. Journal of virology 87: 10126–10138. 23843639 10.1128/JVI.01671-13 PMC3754017

[30] Kuga T, Nozaki N, Matsushita K, Nomura F, Tomonaga T (2010) Phosphorylation statuses at different residues of lamin B2, B1, and A/C dynamically and independently change throughout the cell cycle. Experimental cell research 316: 2301–2312. 20580708 10.1016/j.yexcr.2010.05.017

[31] Gershburg E, Raffa S, Torrisi MR, Pagano JS (2007) Epstein-Barr virus-encoded protein kinase (BGLF4) is involved in production of infectious virus. J Virol 81: 5407–5412. 17360761 10.1128/JVI.02398-06 PMC1900237

[32] Murata T, Isomura H, Yamashita Y, Toyama S, Sato Y, et al (2009) Efficient production of infectious viruses requires enzymatic activity of Epstein-Barr virus protein kinase. Virology 389: 75–81. 19427010 10.1016/j.virol.2009.04.007

[33] Wang JT, Chuang YC, Chen KL, Lu CC, Doong SL, et al (2010) Characterization of Epstein-Barr virus BGLF4 kinase expression control at the transcriptional and translational levels. J Gen Virol 91: 2186–2196. 20444992 10.1099/vir.0.019729-0

[34] Wang JT, Yang PW, Lee CP, Han CH, Tsai CH, et al (2005) Detection of Epstein-Barr virus BGLF4 protein kinase in virus replication compartments and virus particles. J Gen Virol 86: 3215–3225. 16298966 10.1099/vir.0.81313-0

[35] Sugimoto A, Sato Y, Kanda T, Murata T, Narita Y, et al (2013) Different distributions of Epstein-Barr virus early and late gene transcripts within viral replication compartments. Journal of virology 87: 6693–6699. 23552415 10.1128/JVI.00219-13 PMC3676136

[36] Yang PW, Chang SS, Tsai CH, Chao YH, Chen MR (2008) Effect of phosphorylation on the transactivation activity of Epstein-Barr virus BMRF1, a major target of the viral BGLF4 kinase. J Gen Virol 89: 884–895. 18343828 10.1099/vir.0.83546-0

[37] Asai R, Kato A, Kawaguchi Y (2009) Epstein-Barr virus protein kinase BGLF4 interacts with viral transactivator BZLF1 and regulates its transactivation activity. J Gen Virol 90: 1575–1581. 19321754 10.1099/vir.0.010462-0

[38] Yue W, Gershburg E, Pagano JS (2005) Hyperphosphorylation of EBNA2 by Epstein-Barr virus protein kinase suppresses transactivation of the LMP1 promoter. J Virol 79: 5880–5885. 15827205 10.1128/JVI.79.9.5880-5885.2005 PMC1082719

[39] Kato K, Yokoyama A, Tohya Y, Akashi H, Nishiyama Y, et al (2003) Identification of protein kinases responsible for phosphorylation of Epstein-Barr virus nuclear antigen leader protein at serine-35, which regulates its coactivator function. J Gen Virol 84: 3381–3392. 14645919 10.1099/vir.0.19454-0

[40] Wang JT, Doong SL, Teng SC, Lee CP, Tsai CH, et al (2009) Epstein-Barr virus BGLF4 kinase suppresses the interferon regulatory factor 3 signaling pathway. J Virol 83: 1856–1869. 19052084 10.1128/JVI.01099-08 PMC2643756

[41] Wang JT, Chang LS, Chen CJ, Doong SL, Chang CW, et al (2013) Glycogen synthase kinase 3 negatively regulates IFN regulatory factor 3 transactivation through phosphorylation at its linker region. Innate immunity 20 (1) 78–87. 23685991 10.1177/1753425913485307

[42] Chang LS, Wang JT, Doong SL, Lee CP, Chang CW, et al (2012) Epstein-Barr Virus BGLF4 Kinase Down-regulates NF-kappaB Transactivation through Phosphorylation of Coactivator UXT. Journal of virology 86: 12176–12186. 22933289 10.1128/JVI.01918-12 PMC3486492

[43] Mounce BC, Mboko WP, Bigley TM, Terhune SS, Tarakanova VL (2013) A conserved gammaherpesvirus protein kinase targets histone deacetylases 1 and 2 to facilitate viral replication in primary macrophages. Journal of virology 87: 7314–7325. 23616648 10.1128/JVI.02713-12 PMC3700300

[44] Li R, Zhu J, Xie Z, Liao G, Liu J, et al (2011) Conserved Herpesvirus Kinases Target the DNA Damage Response Pathway and TIP60 Histone Acetyltransferase to Promote Virus Replication. Cell host & microbe 10: 390–400. 22018239 10.1016/j.chom.2011.08.013 PMC3253558

[45] Li R, Wang L, Liao G, Guzzo CM, Matunis MJ, et al (2012) SUMO binding by the Epstein-Barr virus protein kinase BGLF4 is crucial for BGLF4 function. Journal of virology 86: 5412–5421. 22398289 10.1128/JVI.00314-12 PMC3347263

[46] Hagemeier SR, Barlow EA, Meng Q, Kenney SC (2012) The cellular ataxia telangiectasia-mutated kinase promotes epstein-barr virus lytic reactivation in response to multiple different types of lytic reactivation-inducing stimuli. Journal of virology 86: 13360–13370. 23015717 10.1128/JVI.01850-12 PMC3503132

[47] Feederle R, Mehl-Lautscham AM, Bannert H, Delecluse HJ (2009) The Epstein-Barr virus protein kinase BGLF4 and the exonuclease BGLF5 have opposite effects on the regulation of viral protein production. J Virol 83: 10877–10891. 19710145 10.1128/JVI.00525-09 PMC2772808

[48] Lee CP, Chen MR (2010) Escape of herpesviruses from the nucleus. Reviews in medical virology 20: 214–230. 20069615 10.1002/rmv.643

[49] Rowe M, Glaunsinger B, van Leeuwen D, Zuo J, Sweetman D, et al (2007) Host shutoff during productive Epstein-Barr virus infection is mediated by BGLF5 and may contribute to immune evasion. Proc Natl Acad Sci U S A 104: 3366–3371. 17360652 10.1073/pnas.0611128104 PMC1805610

[50] Countryman J, Gradoville L, Bhaduri-McIntosh S, Ye J, Heston L, et al (2009) Stimulus duration and response time independently influence the kinetics of lytic cycle reactivation of Epstein-Barr virus. Journal of virology 83: 10694–10709. 19656890 10.1128/JVI.01172-09 PMC2753116

[51] Ye J, Gradoville L, Daigle D, Miller G (2007) De novo protein synthesis is required for lytic cycle reactivation of Epstein-Barr virus, but not Kaposi's sarcoma-associated herpesvirus, in response to histone deacetylase inhibitors and protein kinase C agonists. Journal of virology 81: 9279–9291. 17596302 10.1128/JVI.00982-07 PMC1951462

[52] Gershburg E, Pagano JS (2002) Phosphorylation of the Epstein-Barr virus (EBV) DNA polymerase processivity factor EA-D by the EBV-encoded protein kinase and effects of the L-riboside benzimidazole 1263W94. J Virol 76: 998–1003. 11773375 10.1128/JVI.76.3.998-1003.2002 PMC135851

[53] Carrera AC, Alexandrov K, Roberts TM (1993) The conserved lysine of the catalytic domain of protein kinases is actively involved in the phosphotransfer reaction and not required for anchoring ATP. Proc Natl Acad Sci U S A 90: 442–446. 8421674 10.1073/pnas.90.2.442 PMC45679

[54] El-Guindy A, Ghiassi-Nejad M, Golden S, Delecluse HJ, Miller G (2013) Essential role of rta in lytic DNA replication of epstein-barr virus. J Virol 87: 208–223. 23077295 10.1128/JVI.01995-12 PMC3536415

[55] Neuhierl B, Delecluse HJ (2006) The Epstein-Barr virus BMRF1 gene is essential for lytic virus replication. Journal of virology 80: 5078–5081. 16641300 10.1128/JVI.80.10.5078-5081.2006 PMC1472063

[56] Leng N, Dawson JA, Thomson JA, Ruotti V, Rissman AI, et al (2013) EBSeq: an empirical Bayes hierarchical model for inference in RNA-seq experiments. Bioinformatics 29: 1035–1043. 23428641 10.1093/bioinformatics/btt087 PMC3624807

[57] Li B, Dewey CN (2011) RSEM: accurate transcript quantification from RNA-Seq data with or without a reference genome. BMC bioinformatics 12: 323. 21816040 10.1186/1471-2105-12-323 PMC3163565

[58] Yuan J, Cahir-McFarland E, Zhao B, Kieff E (2006) Virus and cell RNAs expressed during Epstein-Barr virus replication. Journal of virology 80: 2548–2565. 16474161 10.1128/JVI.80.5.2548-2565.2006 PMC1395376

[59] Loesing JB, Di Fiore S, Ritter K, Fischer R, Kleines M (2009) Epstein-Barr virus BDLF2-BMRF2 complex affects cellular morphology. The Journal of general virology 90: 1440–1449. 19264620 10.1099/vir.0.009571-0

[60] Johannsen E, Luftig M, Chase MR, Weicksel S, Cahir-McFarland E, et al (2004) Proteins of purified Epstein-Barr virus. Proc Natl Acad Sci U S A 101: 16286–16291. 15534216 10.1073/pnas.0407320101 PMC528973

[61] Beisser PS, Verzijl D, Gruijthuijsen YK, Beuken E, Smit MJ, et al (2005) The Epstein-Barr virus BILF1 gene encodes a G protein-coupled receptor that inhibits phosphorylation of RNA-dependent protein kinase. Journal of virology 79: 441–449. 15596837 10.1128/JVI.79.1.441-449.2005 PMC538699

[62] O'Grady T, Cao S, Strong MJ, Concha M, Wang X, et al (2014) Global Bidirectional Transcription of the Epstein-Barr Virus Genome during Reactivation. Journal of virology 88: 1604–1616. 24257595 10.1128/JVI.02989-13 PMC3911580

[63] Chen MR, Hsu TY, Chen JY, Yang CS (1990) Molecular characterization of a cDNA clone encoding the Epstein-Barr virus (EBV) DNase. J Virol Methods 29: 127–141. 2176660 10.1016/0166-0934(90)90107-q

[64] Haque M, Wang V, Davis DA, Zheng ZM, Yarchoan R (2006) Genetic organization and hypoxic activation of the Kaposi's sarcoma-associated herpesvirus ORF34-37 gene cluster. J Virol 80: 7037–7051. 16809309 10.1128/JVI.00553-06 PMC1489055

[65] Haque M, Kousoulas KG (2013) The Kaposi's sarcoma-associated herpesvirus ORF34 protein binds to HIF-1alpha and causes its degradation via the proteasome pathway. Journal of virology 87: 2164–2173. 23221556 10.1128/JVI.02460-12 PMC3571462

[66] Francis AL, Gradoville L, Miller G (1997) Alteration of a single serine in the basic domain of the Epstein-Barr virus ZEBRA protein separates its functions of transcriptional activation and disruption of latency. J Virol 71: 3054–3061. 9060666 10.1128/jvi.71.4.3054-3061.1997 PMC191435

[67] Feederle R, Kost M, Baumann M, Janz A, Drouet E, et al (2000) The Epstein-Barr virus lytic program is controlled by the co-operative functions of two transactivators. Embo J 19: 3080–3089. 10856251 10.1093/emboj/19.12.3080 PMC203345

[68] Delecluse HJ, Hilsendegen T, Pich D, Zeidler R, Hammerschmidt W (1998) Propagation and recovery of intact, infectious Epstein-Barr virus from prokaryotic to human cells. Proc Natl Acad Sci U S A 95: 8245–8250. 9653172 10.1073/pnas.95.14.8245 PMC20961

[69] Pearson GR, Vroman B, Chase B, Sculley T, Hummel M, et al (1983) Identification of polypeptide components of the Epstein-Barr virus early antigen complex with monoclonal antibodies. J Virol 47: 193–201. 6306272 10.1128/jvi.47.1.193-201.1983 PMC255226

[70] Song Y, Nagy M, Ni W, Tyagi NK, Fenton WA, et al (2013) Molecular chaperone Hsp110 rescues a vesicle transport defect produced by an ALS-associated mutant SOD1 protein in squid axoplasm. Proceedings of the National Academy of Sciences of the United States of America 110: 5428–5433. 23509252 10.1073/pnas.1303279110 PMC3619309

[71] Trapnell C, Pachter L, Salzberg SL (2009) TopHat: discovering splice junctions with RNA-Seq. Bioinformatics 25: 1105–1111. 19289445 10.1093/bioinformatics/btp120 PMC2672628

[72] Langmead B, Salzberg SL (2012) Fast gapped-read alignment with Bowtie 2. Nature methods 9: 357–359. 22388286 10.1038/nmeth.1923 PMC3322381

[73] Baer R, Bankier AT, Biggin MD, Deininger PL, Farrell PJ, et al (1984) DNA sequence and expression of the B95-8 Epstein-Barr virus genome. Nature 310: 207–211. 6087149 10.1038/310207a0

