# Supplementary figures and images for: A Locus Encompassing the Epstein-Barr Virus bglf4 Kinase Regulates Expression of Genes Encoding Viral Structural Proteins
Source: PLoS Pathog. 2014 Aug 28;10(8):e1004307. doi: 10.1371/journal.ppat.1004307 (PMC4148442; doi:10.1371/journal.ppat.1004307)

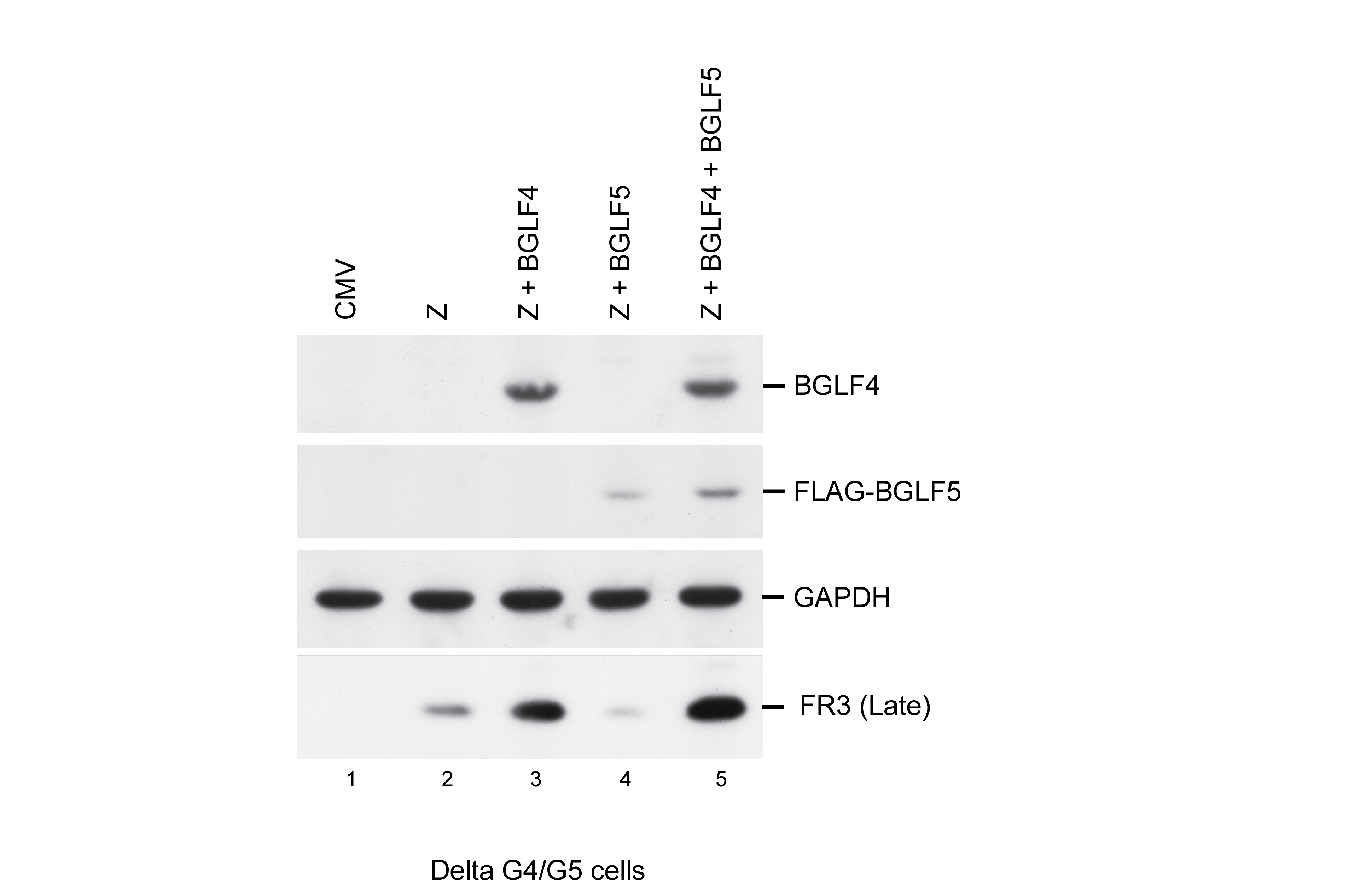

Supplement: Figure S1 — BGLF4 induces expression of the late FR3 protein. In Fig. 1A, we showed that expression of BGLF4, but not BGLF5, in delta G4/G5 cells up-regulated expression of FR3. To confirm this result we repeated the experiment in the same cell line and found that BGLF4 is necessary for efficient expression of the late FR3 protein. Complementation with BGLF5 did not up-regulate the level of FR3 protein (lane 4). (TIF) [file ppat.1004307.s001.tif]

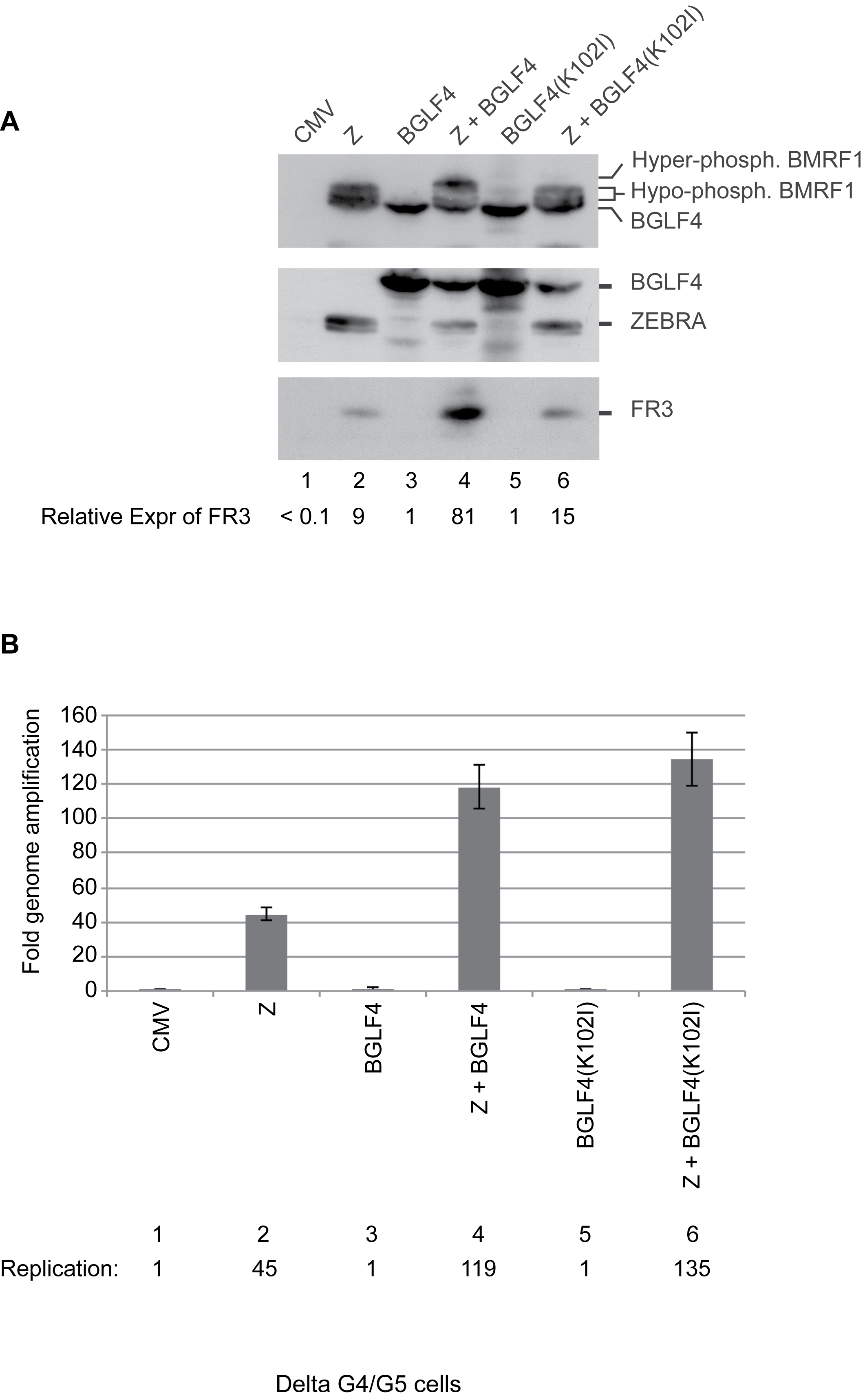

Supplement: Figure S2 — The kinase activity of BGLF4 is necessary for its role in activating late gene expression. (A) Expression of viral lytic proteins in delta G4/G5 cells transfected with empty vector (CMV), ZEBRA (Z), BGLF4, ZEBRA plus BGLF4, kinase dead BGLF4(K102I), and ZEBRA plus BGLF4(K102I). The blot was sequentially reacted with antibodies specific to BFRF3, ZEBRA, BGLF4, and BMRF1. Since BGLF4 and BMRF1 cannot be fully resolved on 10% SDS-PAGE, figure 2A has two panels displaying the BGLF4 protein with and without BMRF1. (B) Fold change in the level of EBV DNA was determined by quantitative PCR in delta G4/G5 cells transfected with the indicated expression vectors relative to empty vector. (TIF) [file ppat.1004307.s002.tif]

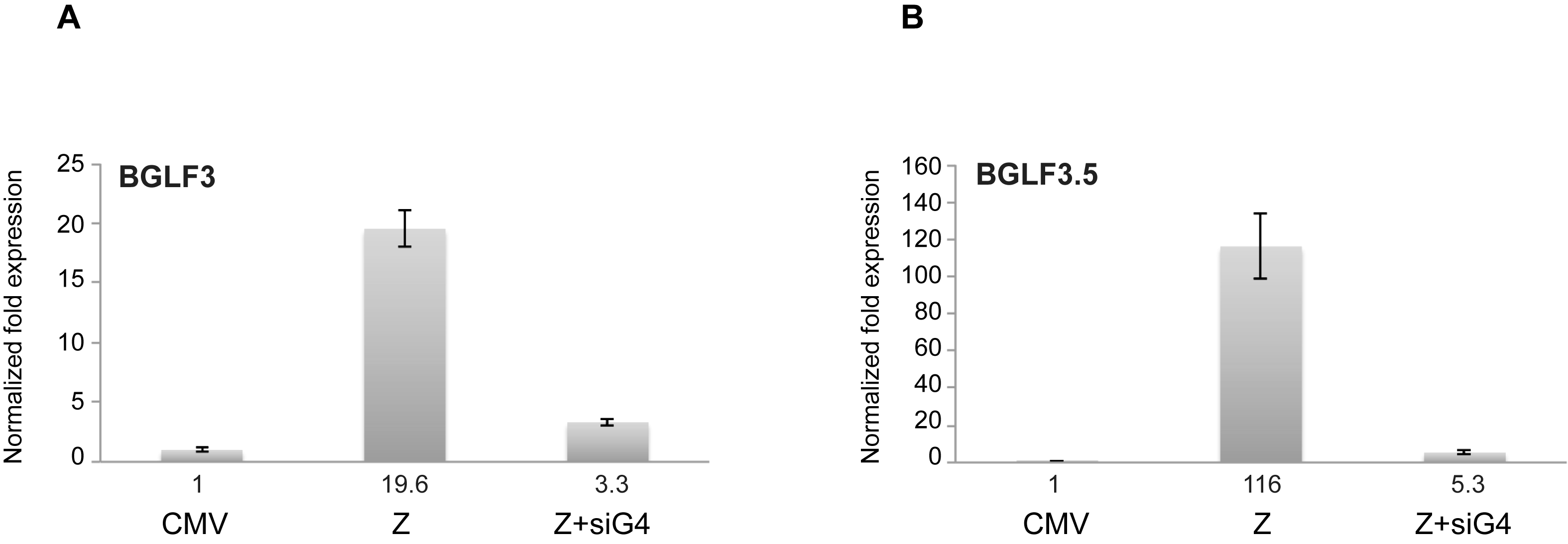

Supplement: Figure S3 — Knockdown of BGLF4 down-regulates expression of BGLF3 and BGLF3.5. Quantitative RT-PCR was used to measure the level of the BGLF3 (A) and BGLF3.5 (B) transcripts in 2089 cells with and without silencing expression of BGLF4. Total cellular RNA was harvested at 48 h after transfection. The relative level of each transcript was calculated as a fold-change relative to empty vector using the ΔΔCT method. (TIF) [file ppat.1004307.s003.tif]

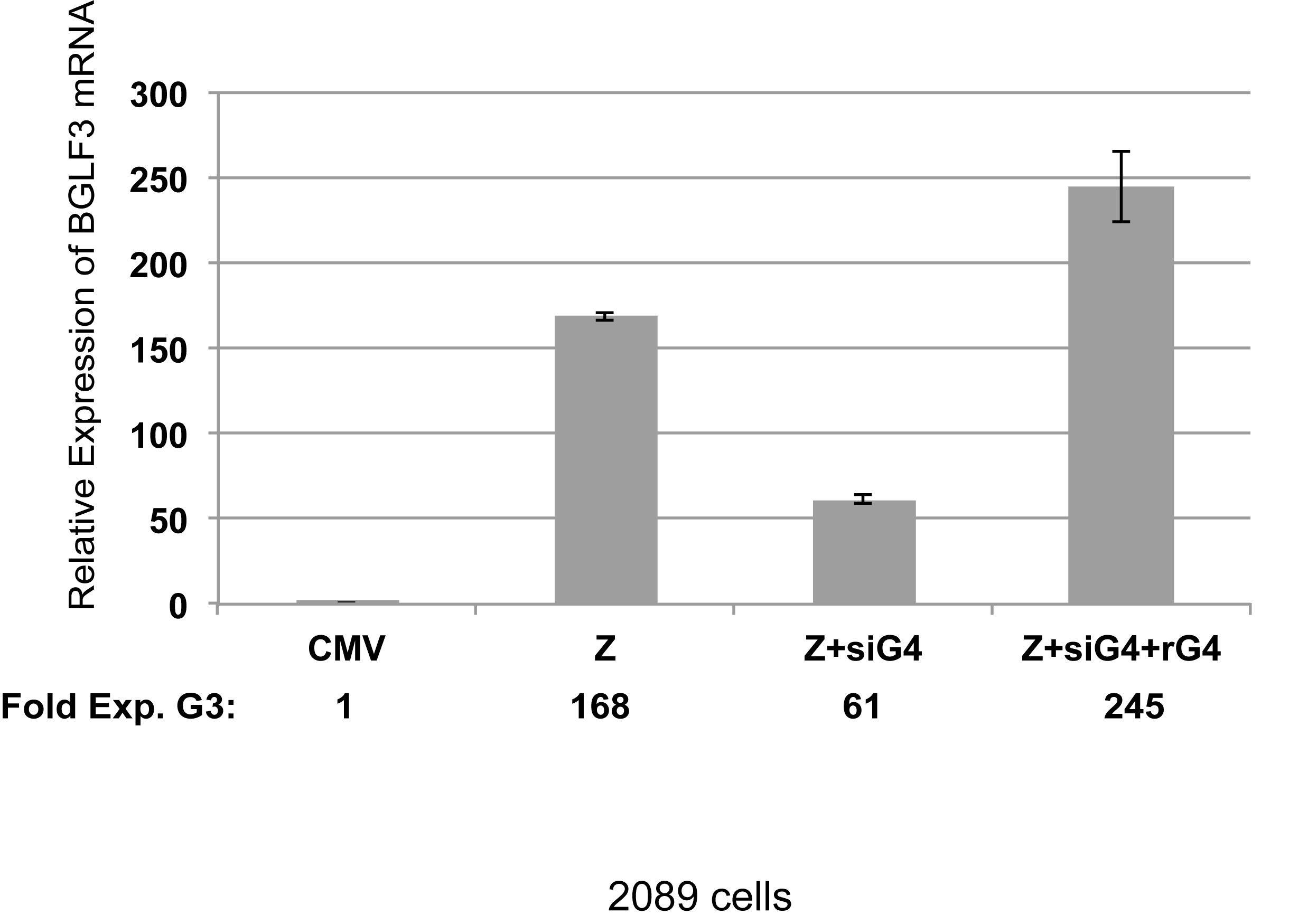

Supplement: Figure S4 — siG4-resistant BGLF4 (rG4) enhances expression of BGLF3. Knockdown of BGLF4 markedly reduced the level of the BGLF3 transcript. Two possible interpretations could be envisioned: i) BGLF4 regulates expression of BGLF3 and ii) BGLF4 and BGLF3 are encoded by the same transcript. To determine if ectopic expression of BGLF4 can up-regulate the level of the BGLF3 mRNA transcribed from the endogenous viral genome, we knocked down the endogenous BGLF4 transcript using siG4 and co-transfected a form of BGLF4 that is resistant to the siRNA. Using RT-qPCR we found that silencing of BGLF4 reduced the level of BGLF3 by 2.8-fold relative to cells expressing ZEBRA. Co-expression of rG4 enhanced expression of BGLF3 by 4-fold compared to cells transfected with ZEBRA plus siG4. This result indicates that BGLF4 has the capacity to induce expression of the endogenous BGLF3 transcript. (TIF) [file ppat.1004307.s004.tif]

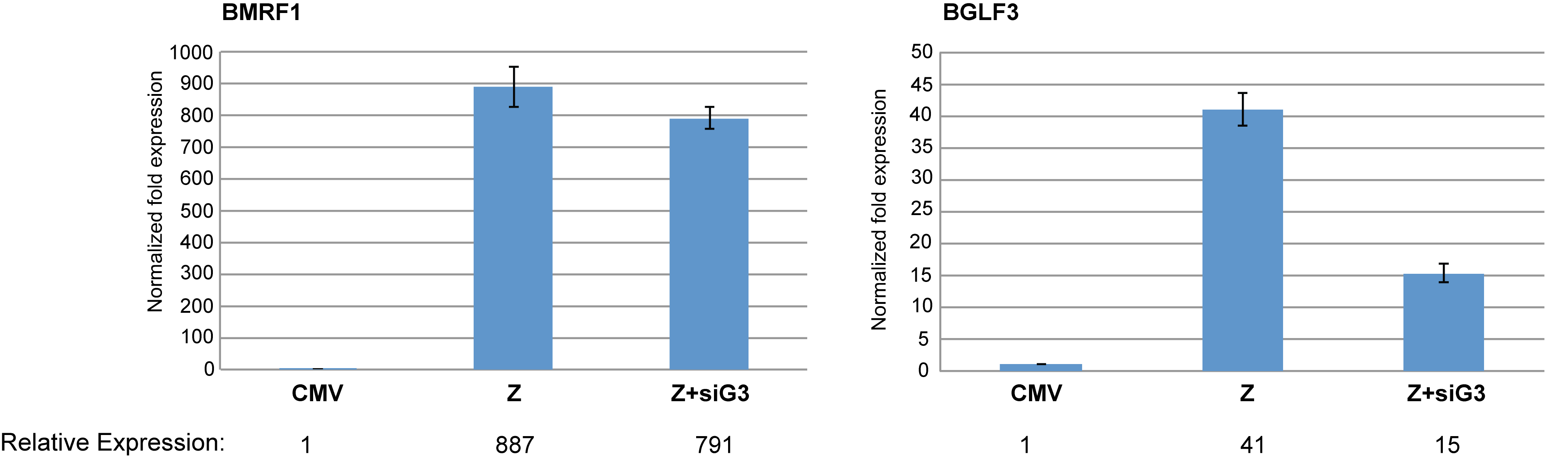

Supplement: Figure S5 — siG3 specifically reduces the level of the BGLF3 transcript. In Fig. 9, we found that siG3 reduced expression of the FR3 late protein without affecting viral DNA replication. To determine if the effect of siRNA to BGLF3 is due to its capacity to target the BGLF3 transcript, we prepared total RNA from samples used in figure 9. Using RT-qPCR we assessed the level of BGLF3 and BMRF1 mRNAs in 2089 cells transfected with wild-type ZEBRA in the absence and presence of siG3. Expression of ZEBRA (Z) stimulated the BMRF1 transcript by 887-fold and the BGLF3 transcript by 41-fold relative to cells transfected with empty vector (CMV). Co-transfection of siG3 had no effect on the level of the BMRF1 transcript but markedly reduced the level of BGLF3 mRNA by 2.7-fold. These results together with data in Fig. 9 demonstrate that the observed effect of siG3 is due to reduction in amount of the BGLF3 transcript. (TIF) [file ppat.1004307.s005.tif]

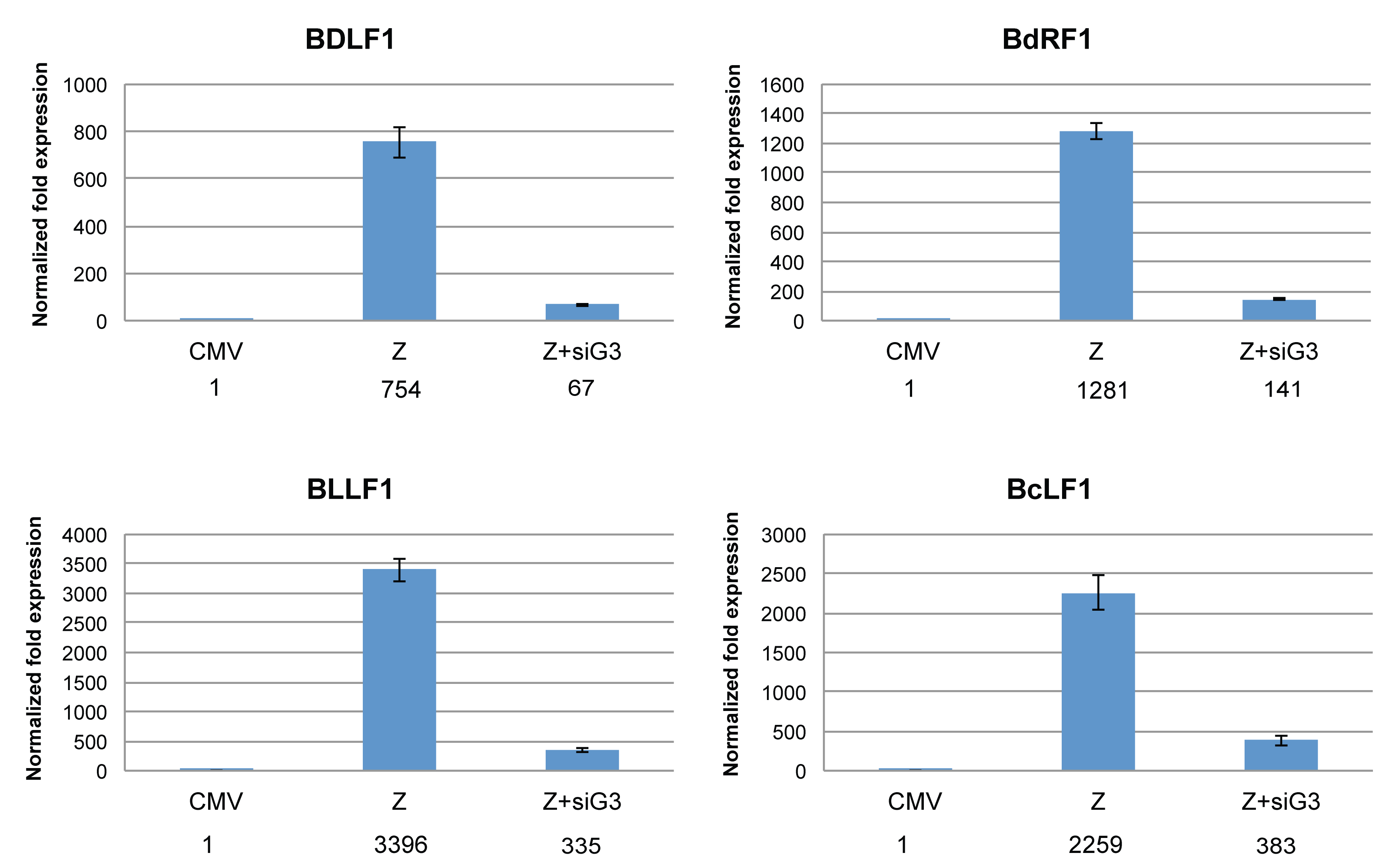

Supplement: Figure S6 — BGLF3 is necessary for expression of late genes. To investigate the effect of silencing BGLF3 on expression of late genes other than bfrf3 (Fig. 9A), we used RT-qPCR to assess the level of four late transcripts, BDLF1, BdRF1, BLLF1, and BcLF1, in the same RNA samples studied in Fig. S5. We found that knockdown of BGLF3 reduced the level of these late transcripts in 2089 cells transfected with ZEBRA plus siG3 relative to cells expressing ZEBRA alone by 11.2, 9.1, 10.1, and 5.9-fold respectively. In the same experiment (Fig. 9), the level of BGLF4 protein was not affected by siG3. These results demonstrate that BGLF3 plays an essential role in regulating expression of late genes during the EBV lytic cycle. (TIF) [file ppat.1004307.s006.tif]

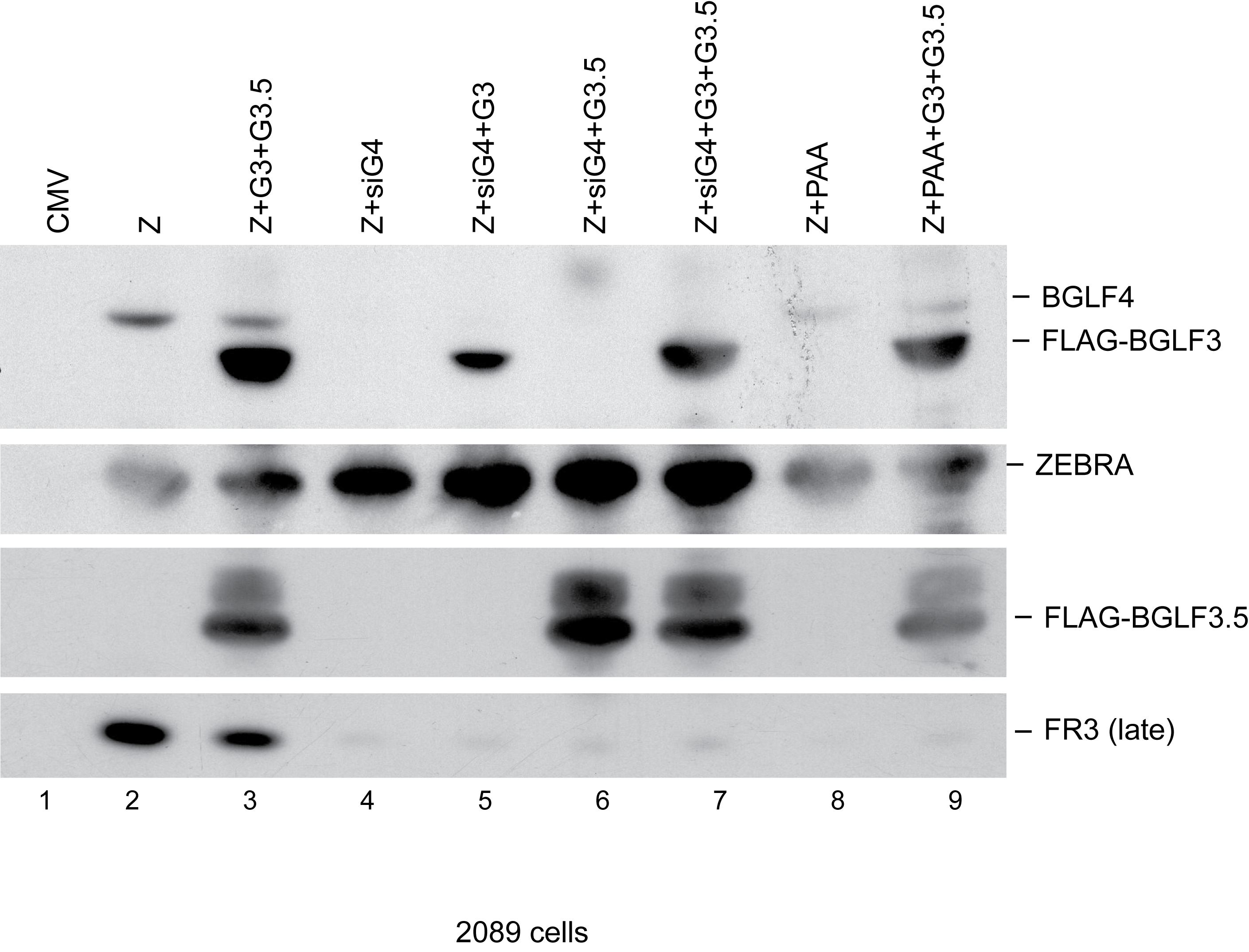

Supplement: Figure S7 — Expression of BGLF3 and BGLF3.5 is not sufficient to activate late gene expression in the absence of BGLF4. We examined whether compensatory expression of BGLF3 and BGLF3.5 in the absence of BGLF4 would rescue expression of the late FR3 protein. 2089 cells were transfected with the indicated expression vectors with and without siG4. Cells were harvested after 48 h and expression of lytic proteins was analyzed by Western blot. We found that knockdown of BGLF4 abolished synthesis of FR3 (compare lane 2 with lane 4). Ectopic expression of BGLF3 and BGLF3.5 in 2089 cells with siG4 failed to restore expression of the late FR3 protein (compare lane 3 with lane 7). This finding indicates that BGLF3 and BGLF3.5 do not substitute for the role of BGLF4 in regulation of late gene expression; BGLF4 has an independent role in activation of late gene expression. (TIF) [file ppat.1004307.s007.tif]
